# Supplementary material for: Phenotypes on demand via switchable target protein degradation in multicellular organisms
Source: Nat Commun. 2016 Jul 22;7:12202. doi: 10.1038/ncomms12202 (PMC4961840; doi:10.1038/ncomms12202)

Supplementary Figure 1 The N-Degron approach. Related to Figure 1. **(a)** N-degron constructs contain a 5' degron cassette starting with a sequence encoding one single ubiquitin (Ub; Ub-fusion technique) followed by mouse dihydrofolate reductase (DHFR) with the first triplet coding for a destabilizing residue (here a bulky hydrophobic amino acid; B). The gene of interest (GOI) is fused to the 3' end of the cassette. **(b)** The N-degron fusion protein consists of an N-terminal Ub (76 amino acids, 8.5 kDa) and the destabilizing residue preceding the temperature-sensitive (ts) variant of mouse DHFR (22 kDa). DHFR contains 16 Lys (K) residues that can be partially exposed to the surface at restrictive temperatures (d). **(c)** The Ub-fusion technique (UFT) is based on the cotranslational deubiquitylation by deubiquitylating enzymes (DUBs) and Ub-specific processing proteases (Ubps) revealing the actual N-degron which is (partially) inactive at permissive temperature (e.g. 23 to 24°C in yeast or mammalian cell culture). **(d)** A shift to restrictive temperature (e.g. 37°C (yeast), 42°C (animal cell culture), or 27 to 29°C using our low-temperature degron in plants and *Drosophila*) promotes $DHFR^{ts}$ flexibility and exposure of internal Lys residues. **(e)** In *S. cerevisiae*, the destabilizing N-terminal residue (B) is recognized by the N-recognin Ubr1 E3 ligase, i.e. an N-end rule pathway recognition component. Uba1 (E1 Ub-activating enzyme) and Ubc2/Rad6 (E2 Ub-conjugating enzyme) prime Ub for transfer to the DHFR moiety of the fusion protein. **(f)** Polyubiquitinylation targets the entire fusion protein for degradation by the 26S proteasome.[1]

Supplementary Figure 1

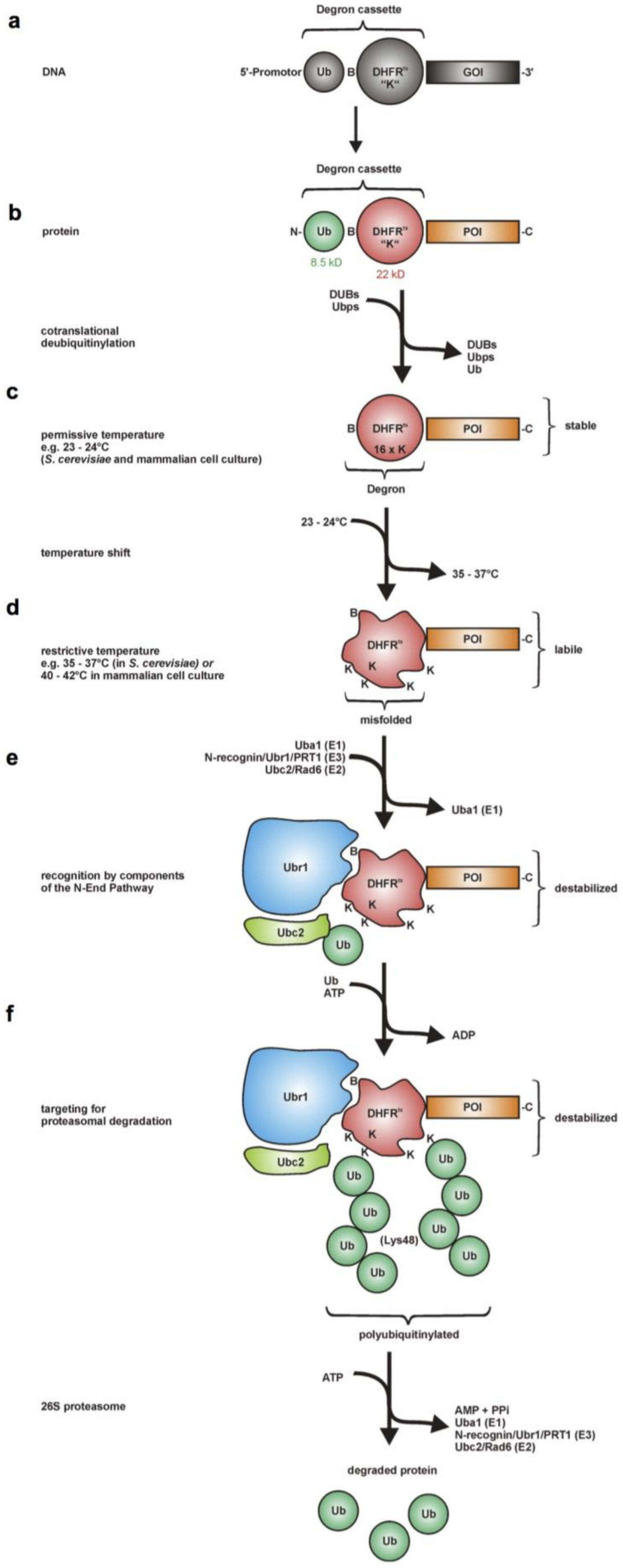

Supplementary Figure 2 Assembly of degron constructs. Related to Figure 1, 2, 3, 4, 4, 6, and 7. Primers used for PCR and cloning as well as sequencing are annotated for better understanding of the degron composition (black arrows). Each degron construct carries a DHFR variant and a triple hemagglutinin epitope (HAT with single HA tags indicated with red arrows). K1, K2, and K3 start with Ub and a destabilizing amino acid residue which was engineered at the junction between Ub and DHFR. **(a)** Degron cassette K1 containing $DHFR^{P67L}$. **(b)** Degron cassette K3 containing $DHFR^{T39A/P67L/E173D}$. **(c)** Degron cassette K2 containing $DHFR^{T39A/E173D}$. **(d)** Degron cassette K4 containing $DHFR^{T39A/E173D}$. K4 serves as control independent of the N-end rule pathway for K2 as it directly starts with Met-DHFR. All shown constructs start with a Gateway attB1 recombination site and end with the protein of interest (POI) and an attB2 recombination site. K1 and K4 contain a NotI restriction site at the HAT–POI junction due to the cloning strategy. **(e)** K1 constructs fused to TTG1, and CO. **(f)** K3 constructs fused to TTG1 and CO and K3:TTG1 without HGSGI linker between the destabilizing N-terminal Phe residue and the DHFR moiety. This linker was also used in the 5' region of the original DHFR containing N-end rule test substrates in Bachmair *et al*. (1989). **(g)** K2 degron constructs. *K2:POI* fusions used in this study (TTG1, CO, GUS, TEV, PAT, and GFP). *K2:GFP* for plants and flies contain a Gateway attB2 recombination site due to the cloning procedure. **(h)** K4 containing constructs fused to TTG1 and CO which serve as controls independent of the N-end rule pathway. **(i)** *S. cerevisiae* degron constructs. The reporter protein is URA3, the constructs contain a wildtype DHFR as stable control which does not respond to high temperatures, or the K1 and K2 cassettes comprising point mutations in the DHFR moiety. K2-URA3 harbors the two point mutations isolated from the yeast mutagenesis screen leading to a lower restrictive temperature compared to the original constructs.[2] All constructs contain the linker used in the plant and fly constructs. Construct design, sequence and map database management done with Vector NTI Advance 10.3.1 (Invitrogen). All con-

structs were assembled as Gateway Entry clones and electronic maps in genbank format are available upon request.

Supplementary Figure 2

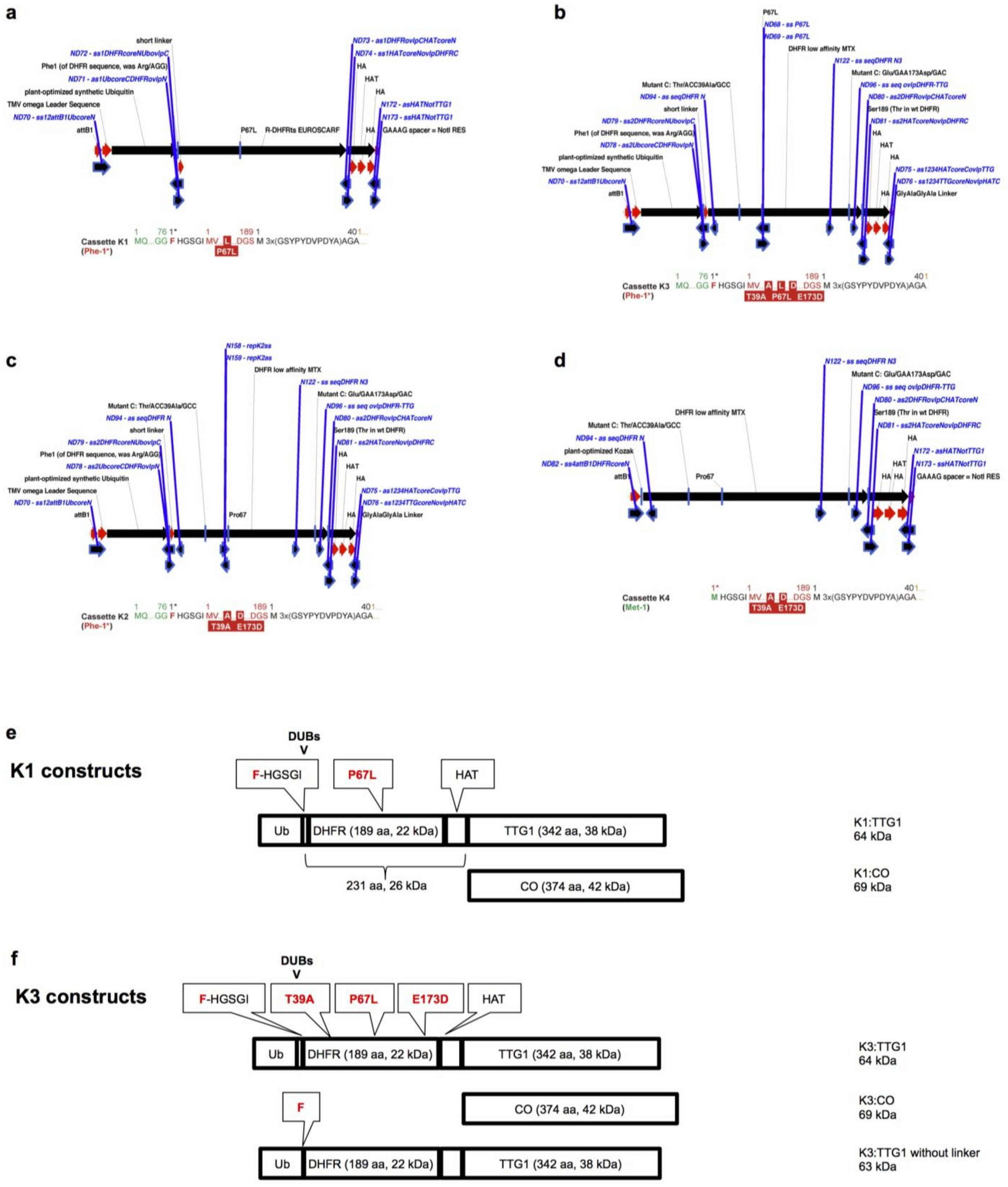

Supplementary Figure 2, continued

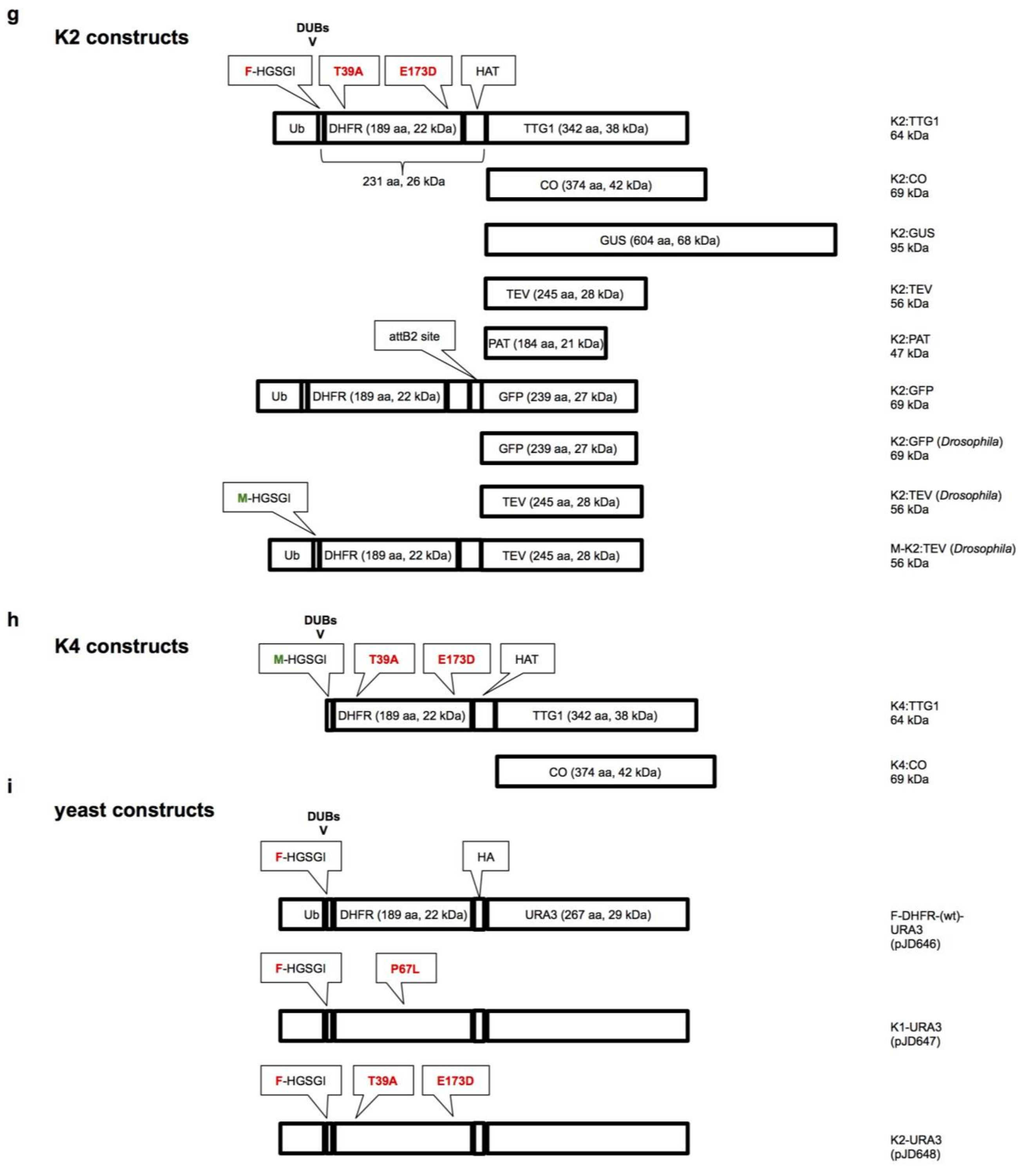

Supplementary Figure 3. Stability of TTG1 and conditional complementation of *ttg1* with various *TTG1-td* variants. Related to Figure 1 and 2. **(a)** Stability of TTG1 depending on the temperature. Western blot of crude extract of a *ttg1 TTG1:HA* line that served as a negative control, MW: 45 kDa. Asterisk: non-specific binding of the anti-HA antibody. **(b)** Conditional complementation of *ttg1* with different *TTG1-td* variants. Related to Figure 1 and 2. To compare phenotypes of plants expressing *K1* to *K4:TTG1*, four-week old *ttg1* plants containing different potential conditional *TTG1-td* rescue constructs were grown at permissive (17°C) or restrictive (29°C) temperature. **(I-IV)** *K1:TTG1*, **(V-VIII)** *K3:TTG1*, **(IX-XII)** *K2:TTG1*, and **(XIII-XVI)** *K4:TTG1*. Same magnifications in: I, V, IX, XII; II, VI, IX, XIII; III, VII, X, XIV; and IV, VIII, XI, XV. Scale bars, I, V, IX, XII, III, VII, X, XIV, 1 cm; II, VI, IX, XIII, IV, VIII, XI, XV, 2 mm. Column two and four show 5X magnified inlets of the first and third columns. Details on constructs used are shown in **Supplementary Figure 2**. **(c) to (g)** Phenotypes of K3:TTG1 containing triple mutant DHFR. (c) Both protein levels of K3:TTG1 and **(d)** K3:TTG1 (without linker) are undetectable at permissive temperatures. **(e)** Transcript levels of transgenic plants as in (c) and (d). The wild type control in (e) is also shown in Supplementary Figure 4c and comes from the same experiment. Plants expressing *K3:TTG1* with **(f)** or without **(g)** linker sequence between N-terminal Phe residue and mouse DHFR of the N-degron moiety fail to develop trichomes at permissive temperature. Scale bar 1 cm. Data were confirmed by analysis of at least three biological replicates. Equal loading was further confirmed by staining of blotted membranes with Ponceau S.

Supplementary Figure 3

a

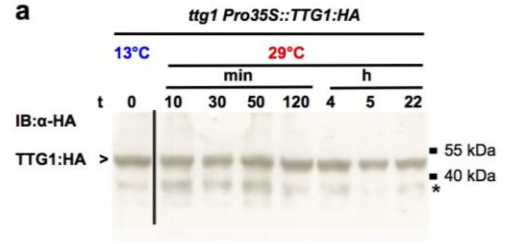

b

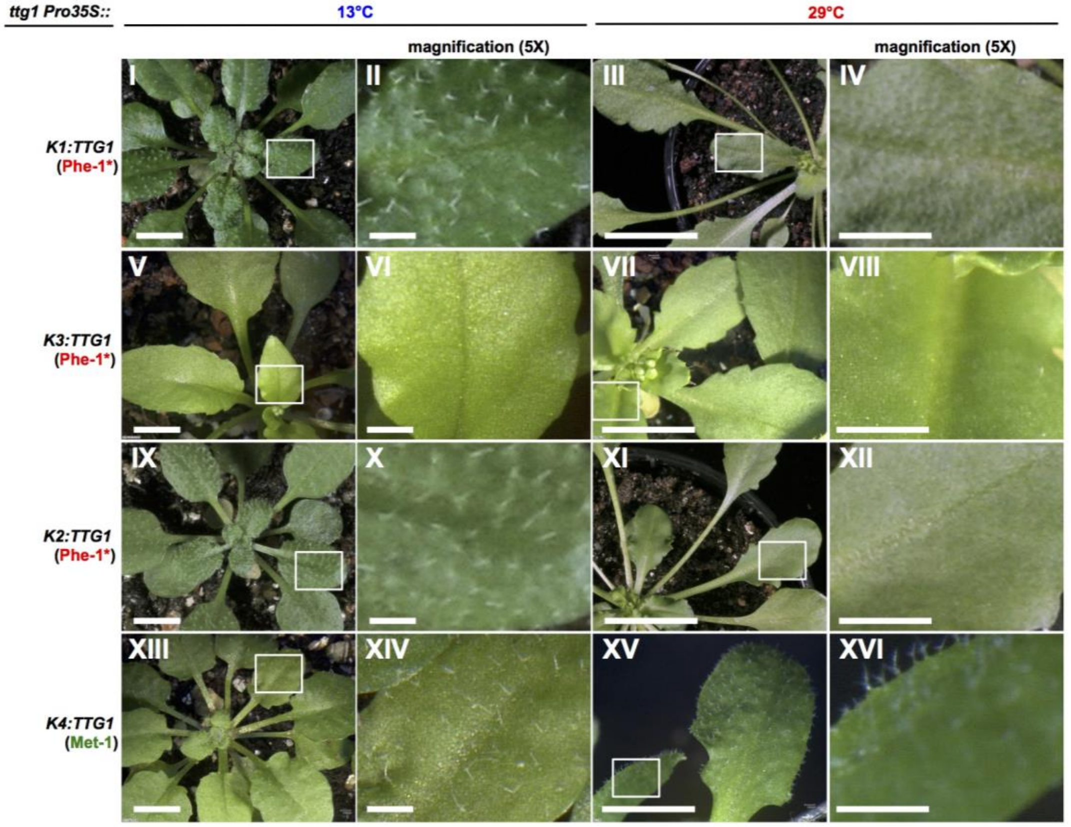

c

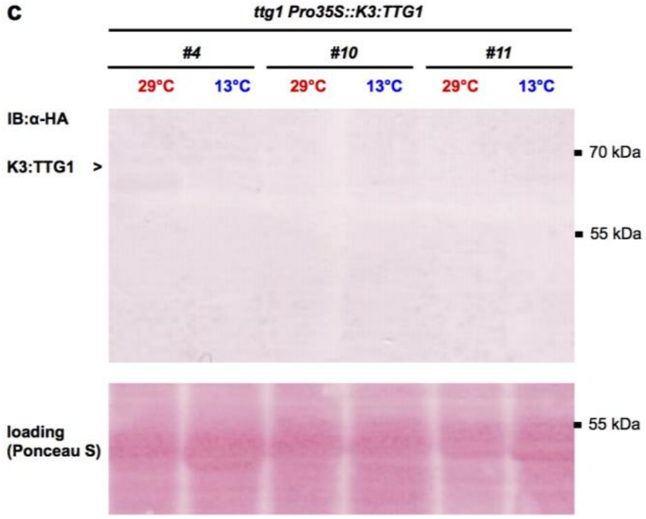

Supplementary Figure 3, continued

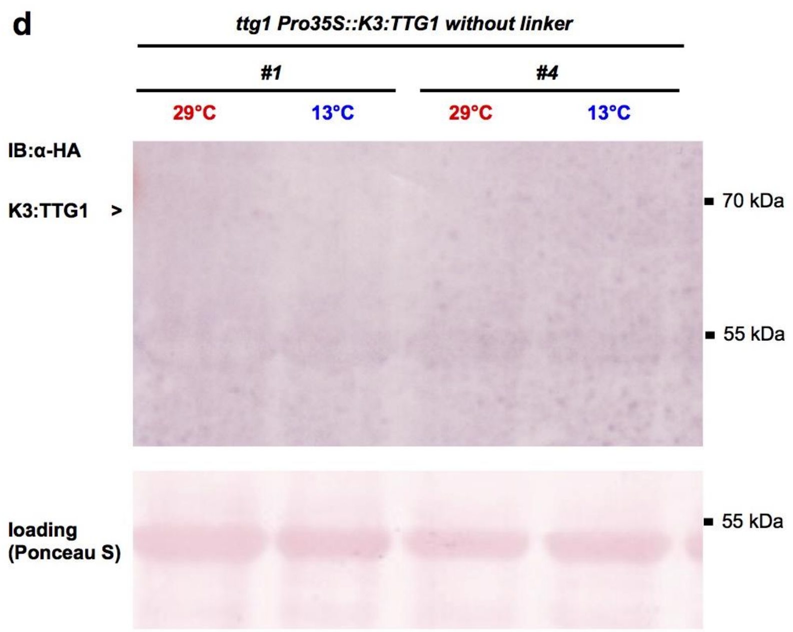

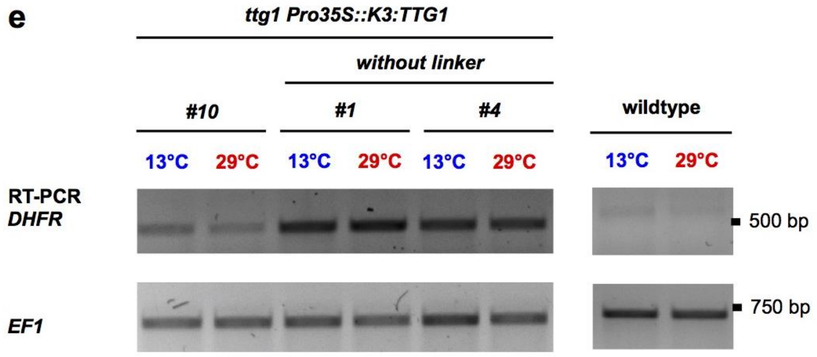

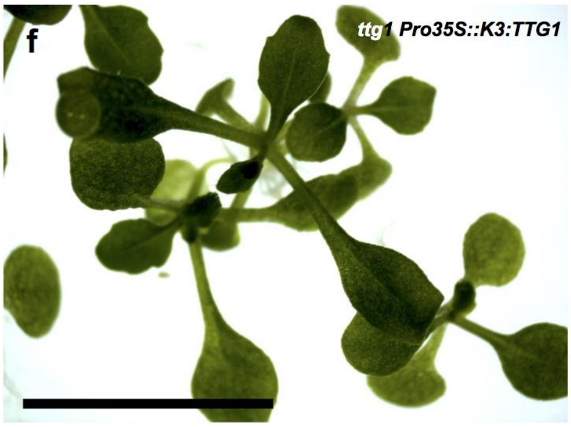

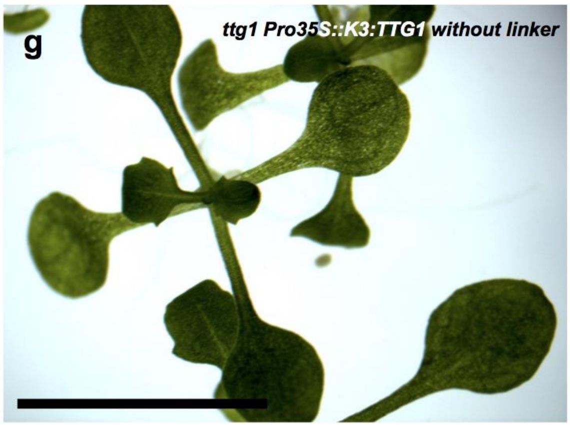

Supplementary Figure 4 Transcript levels and proteasome-dependent degradation of responsive N-degron lines. Related to Figure 1, 2, 3, and 4. **(a) to (c), (e) and (g)** *Arabidopsis* plants were grown aseptically on selective media at constitutively permissive or restrictive standard (LD) conditions. 13-day-old seedlings were harvested and proteins extracted using RIPA buffer for western blot. 40 µg of total protein were loaded per lane and *K2* containing fusion proteins probed with anti-HA antibody. Semi-quantitative RT-PCR was done with an N-degron specific primer set for DHFR. Primers against *ELONGATION FACTOR 1* (*EF1*) were used as housekeeping control. **(c) to (c)** Transcript levels of *K2:TTG1*, *K2:CO*, and *K2:GFP*. #F6, #F41, #R42, and #R45 refers to independent transgenic lines with initiating Phe (F) or Arg (R) residues at the neo-N-terminal after deubiquitination, respectively. The wild type control in (c) is also shown in Supplementary Figure 3e and comes from the same experiment. **(d)** *R*- and *F-K2:GFP* time-course experiments. *K2* cassettes are initiated either with an Arg or a Phe residue. **(e)** Transcript levels of *K2:GUS*. **(f)** Proteasomal degradation of *K2:GUS*. Seedlings were grown in liquid culture for 2 weeks shaking in long-day conditions at 21°C. 48h prior to the treatment experiment flasks containing the seedling were shifted to permissive (13°C) or restrictive (29°C) conditions. At the day of the experiment, seedlings were treated with 50 µM MG132 or a mock treatment (DMSO) for 5 h. Data were confirmed by analysis of two biological replicates. **(g)** Transcript levels of *K2:TEV*. Equal loading was further confirmed by staining of blotted membranes with Ponceau S or with Coomassie Brilliant Blue G225 after immunostaining. Details on constructs used in **Supplementary Figure 2**.

Supplementary Figure 4

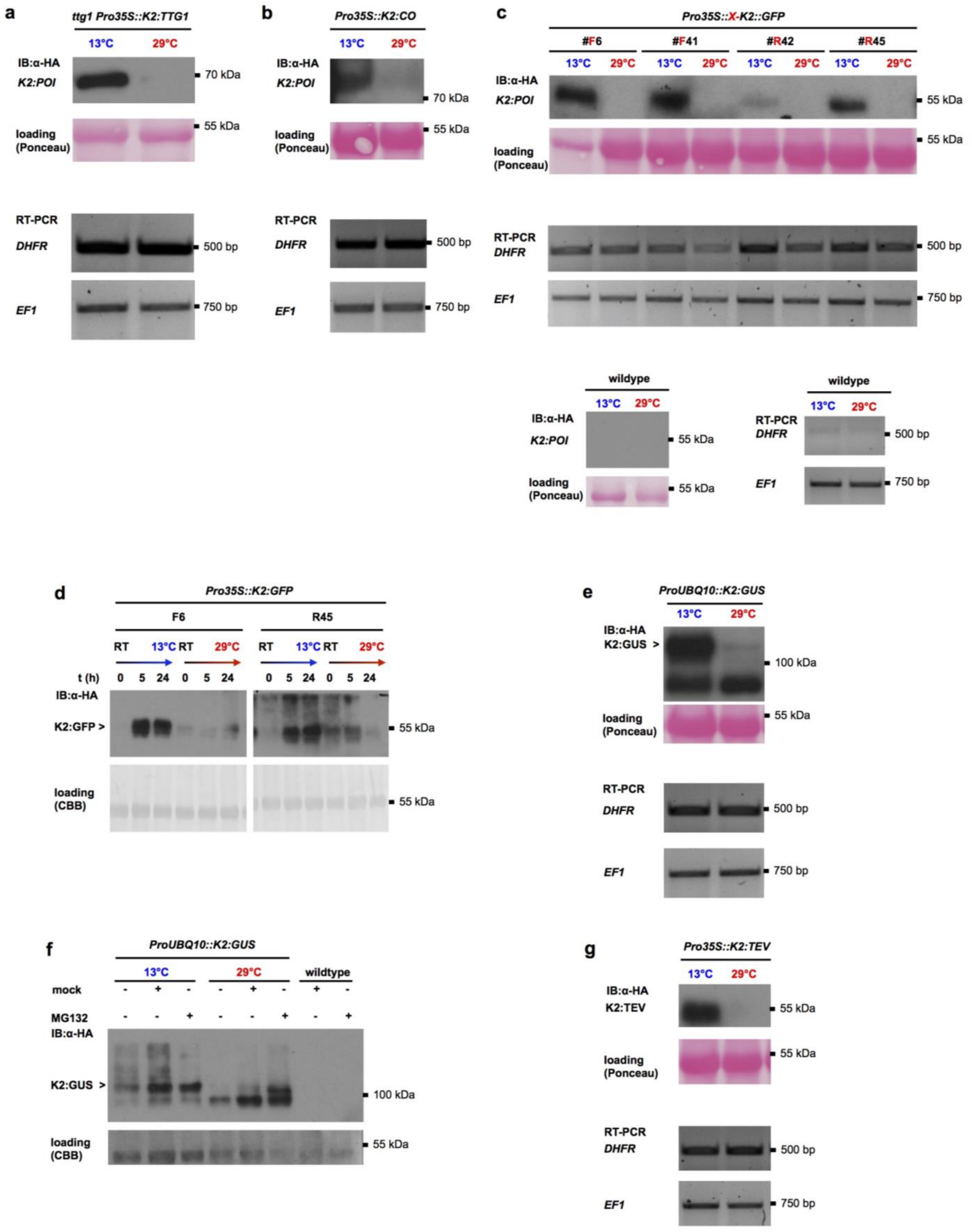

Supplementary Figure 5 Molecular dynamics simulations and modeling of DHFR point mutations of degron cassettes K1, K2, and K3. Related to Figure 1 to 7. Differences of the RMSD fluctuations of the amino acid residues of DHFR. Positive RMSD fluctuations indicate an increased flexibility at the respective domain or residue in comparison to the wildtype whereas negative values hint towards a more rigid state. (**a**) Differences between the K1 mutant and the wild type enzyme; (**b**) between the K2 mutant and the wild type; and (**c**) between the K3 mutant and the wild type enzyme. Residues with predicted higher flexibility potentially leading to a better accessibility for ubiquitination are indicated: Arg29, Lys33, Lys69, and Lys174. See also (**e) to (i)**. (**d**) Disorder, hydropathy, and secondary structure of murine DHFR deduced from PDB ID 1U70. Possible DHFR point mutations are located in helices 1 (T39A) and 3 (P67L) as well as strands 10 (E173D). Information retrieved from RCSB Protein Data Bank, 1U70 Sequence Report at http://www.rcsb.org and UniProt at http://www.uniprot.org, UniProtKB: P00375. **(e) to (i)** Modeling of the DHFR of degron cassettes K1, K2, and K3. To predict structural deviations in the DHFR sequence, the three different point mutations used in the DHFR variants were modeled onto the wild type structure (PDB ID: 1U70). In the graphical representations, the wild type amino acid residues are in grey (atom-type colored), the corresponding wild type amino acid exchanges caused by point mutations in green and the residues of the mutated enzymes that alter their conformation are highlighted in magenta. **(e)** T39A (K2 and K3) and side chain conformations in the close vicinity of the point of mutation. **(f)** T39A (K2) in a simulation where Pro67 (K1 and K3) is unchanged. After removal of the co-crystallized ligand, a neighboring residue, **Lys69**, becomes flexible and potentially more accessible for ubiquitination. **(g)** P67L (K1 and K3) in the triple mutated K3 variant. Molecular dynamics simulations show that **Lys69** becomes more flexible and potentially more accessible for ubiquitination (**a to c**). **(h)** E173D (K2 and K3) in the double mutated K2 variant. The mutation causes a higher flexibility and accessibility of **Lys174**, accompanied with conformational change of side chains of **Arg29** and **Lys33**

like in K3. **(i)** E173D in the triple mutated K3 variant. This mutation also causes higher flexibility of and a conformational change of Arg29 and Lys33 like in K2 (**a to c**). The sequence, the PDB is associated with, lacks the initiating Met. Therefore, in the images (and the PDB file), Pro67 has the ID 66, Thr39 the ID 38, and Glu173 the ID 172. Information from RCSB Protein Data Bank, 1U70 Sequence Report; http://www.rcsb.org.

Supplementary Figure 5

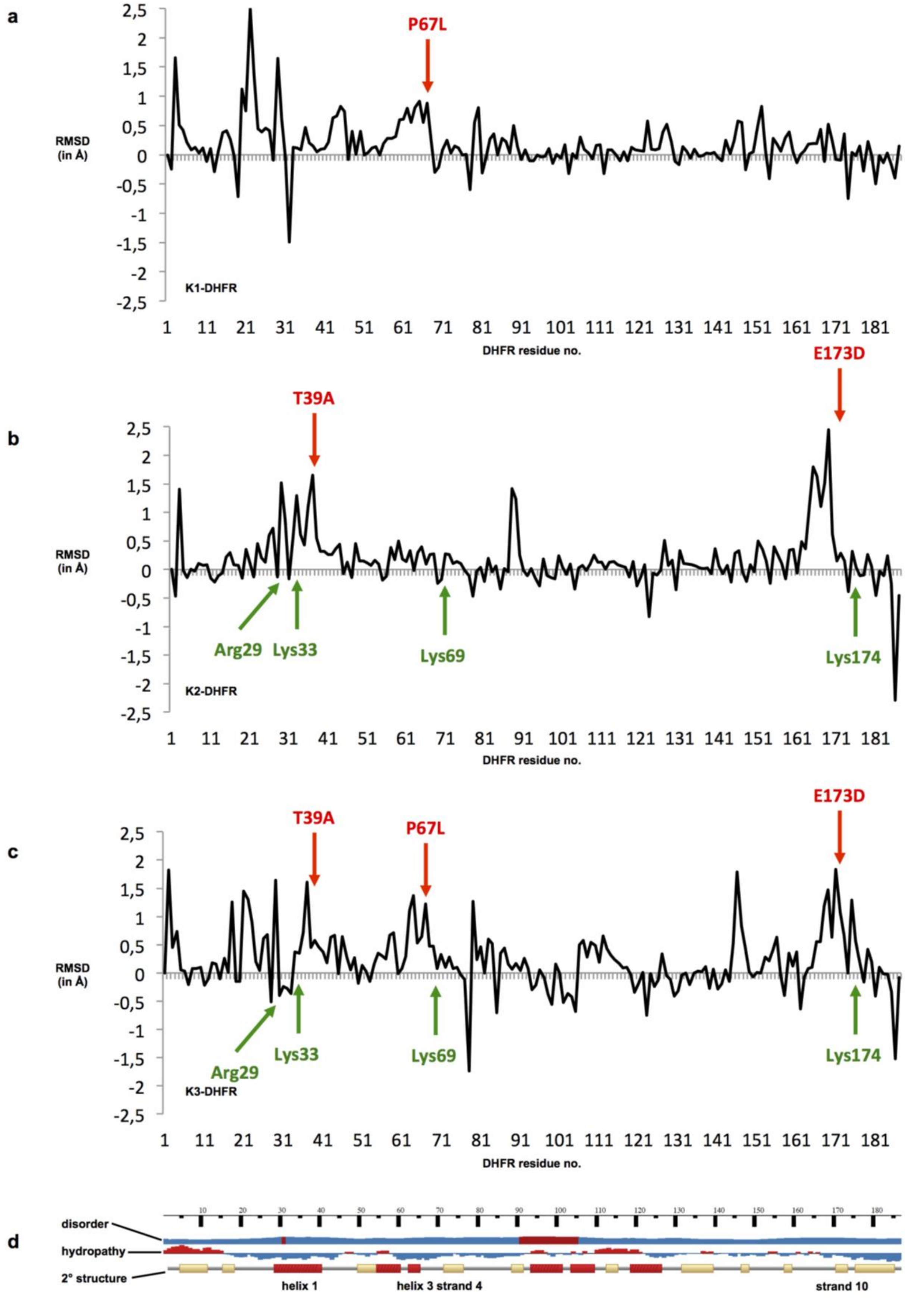

Supplementary Figure 5, continued

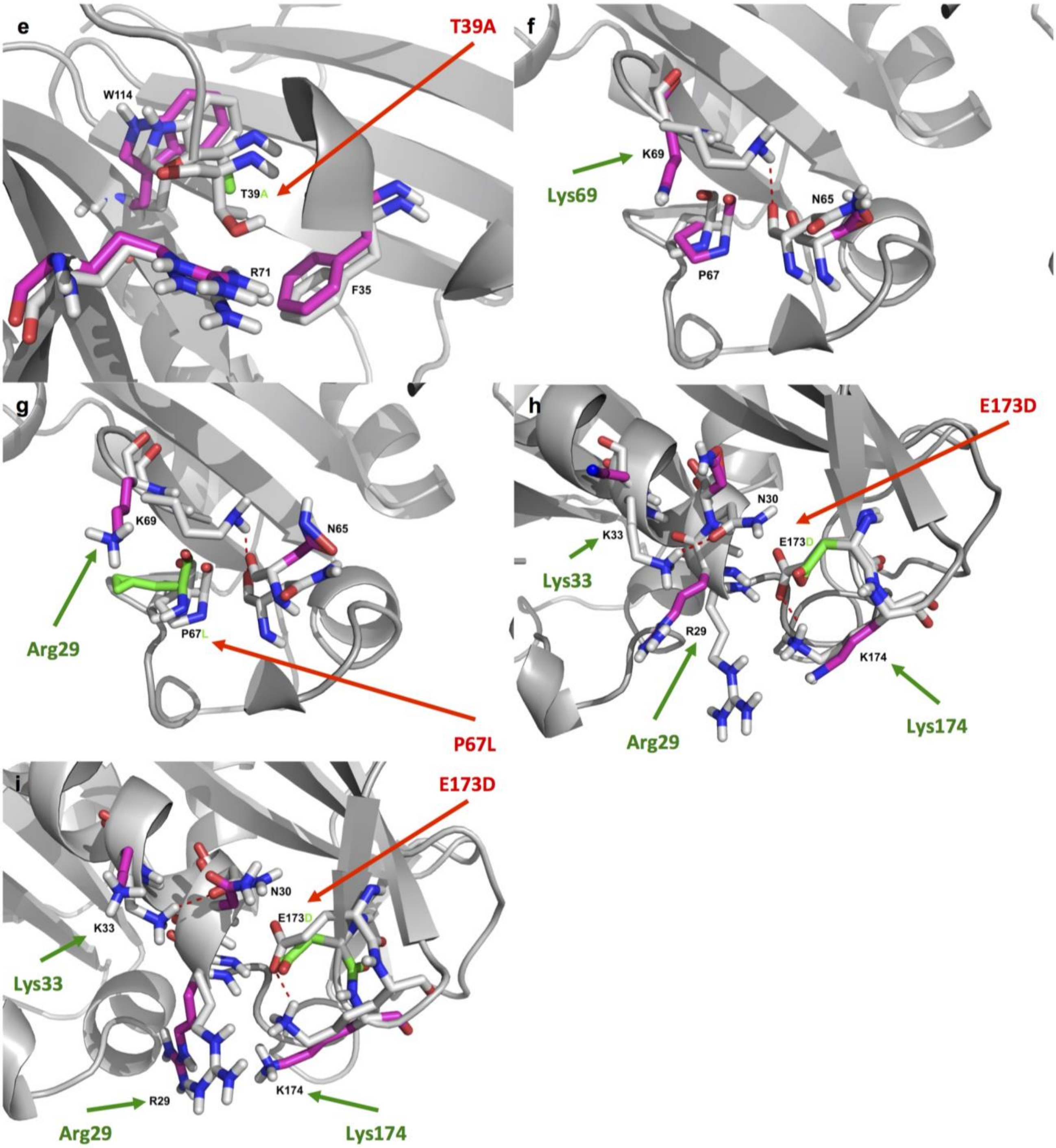

Supplementary Figure 6 Developmental time-course of *K2:CO*. Related to Figure 3. Time-course to monitor onset of flowering in *K2:CO* versus wildtype. Plants were grown under long-day conditions until flower buds emerged. *K2:CO* plants grown under permissive conditions started bolting after 40 days and flowered after 45 days. The wildtype control started flowering about 2 weeks after the *K2:CO* plants. We did not observe any distinguishable phenotype between wildtype and *K2:CO* plants under restrictive conditions where all plants began to flower after 2.5 weeks.

Supplementary Figure 6

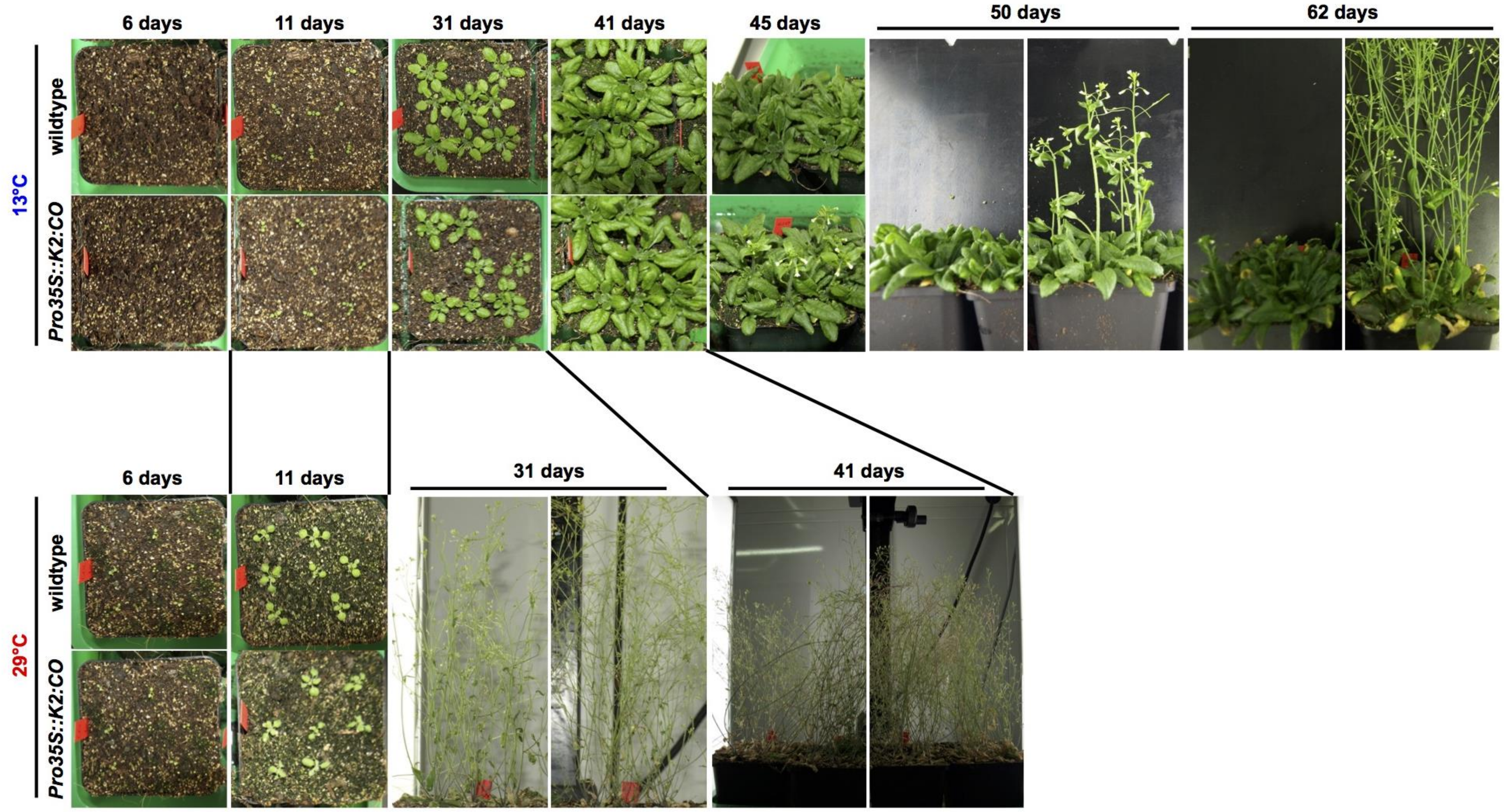

Supplementary Figure 7 Mass spectrometry of *K2:GUS*. Related to Figure 4. *ProUBQ10::K2:GUS* plants were grown under aseptic conditions at permissive temperature (13°C) for 2 weeks. **(a)** proteins extracted and *K2:GUS* immunoprecipitated using an anti-DHFR antibody and immunostained with anti-HA antibody. M: marker. **(b)** Silver stained gel of *K2:GUS* immunoprecipitation. The three bands indicated were isolated and subjected to liquid chromatography mass spectrometry (LC-MS). M: marker. **(c)** Sequence coverage of MS analysis: *K2:GUS* sequence containing F-HGSGI-DHFR (K2 part, underlined), HAT with linkers (blue), and GUS (black only). Detected peptides are highlighted in red. Details on constructs used in **Supplementary Figure 2**.

Supplementary Figure 7

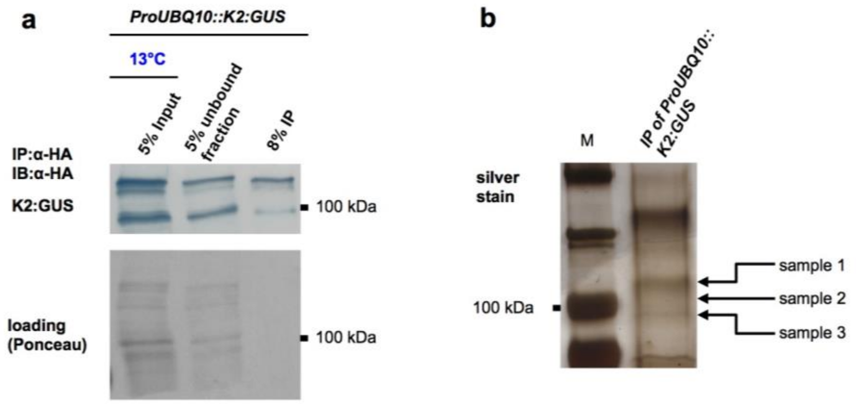

c

F-HGSGI | E173D | T39A | HAT | GUS

Sample 1

FHGSGIMVRP LNCIVAVSQN MGIGKNGDLP WPPLRNEFKY FQRMATTSSV EGKQNLVIMG RKTWFSIPEK NRPLKDRINI
VLSRELKEPP RGAHFLAKSL DDALRLIEQP ELASKVDMVW IVGGSSVYQE AMNQPGHLRL FVTRIMQEFE SDTFFPEIDL
GKYKLLPEYP GVLSEVQEDK GIKYKFEVYE KKDGSMGSYP YDVPDYAGYP YDVPDYAGSY PYDVPDYAGA GA...

F-HGSGI, DHFR^{T39A,E173D}, HAT

MLRPVETPTR EIKKLDGLWA FSLDRENCGI DQRWWESALQ ESRAIAVPGS FNDQFADADI RNYAGNVWYQ REVFIPKGWA
GQRIVLRFDA VTHYGKVWVN NQEVMEHQGG YTPFEADVTP YVIAGKSVRI TVCVNNELNW QTIPPGMVIT DENGKKKQSY
FHDFFNYAGI HRSVMLYTTP NTWVDDITVV THVAQDCNHA SVDWQVVANG DVSVELRDAD QQVVATGQGT SGTLQVVNPH
LWQPGEGYLY ELCVTAKSQT ECDIYPLRVG IRSVAVKGQQ FLINHKPFYF TGFGRHEDAD LRGKGFDNVL MVHDHALMDW
IGANSYRTSH YPYAEEMLDW ADEHGIVVID ETAAVGFNLS LGIGFEAGNK PKELYSEEAV NGETQQAHLQ AIKELIARDK
NHPSVVMWSI ANEPDTRPQG AREYFAPLAE ATRKLDPTRP ITCVNVMFCD AHTDTISDLF DVLCLNRYYG WYVQSGDLET
AEKVLEKELL AWQEKLHQPI IITEYGVDTL AGLHSMYTDM WSEEYQCAWL DMYHRVFDRV SAVVGEQVWN FADFATSQGI
LRVGGNKKGI FTRDRKPKSA AFLLQKRWTG MNFGEKPQQG GKQ

GUS

Sample 2

FHGSGIMVRP LNCIVAVSQN MGIGKNGDLP WPPLRNEFKY FQRMATTSSV EGKQNLVIMG RKTWFSIPEK NRPLKDRINI
VLSRELKEPP RGAHFLAKSL DDALRLIEQP ELASKVDMVW IVGGSSVYQE AMNQPGHLRL FVTRIMQEFE SDTFFPEIDL
GKYKLLPEYP GVLSEVQEDK GIKYKFEVYE KKDGSMGSYP YDVPDYAGYP YDVPDYAGSY PYDVPDYAGA GA...
MLRPVETPTR EIKKLDGLWA FSLDRENCGI DQRWWESALQ ESRAIAVPGS FNDQFADADI RNYAGNVWYQ REVFIPKGWA
GQRIVLRFDA VTHYGKVWVN NQEVMEHQGG YTPFEADVTP YVIAGKSVRI TVCVNNELNW QTIPPGMVIT DENGKKKQSY
FHDFFNYAGI HRSVMLYTTP NTWVDDITVV THVAQDCNHA SVDWQVVANG DVSVELRDAD QQVVATGQGT SGTLQVVNPH
LWQPGEGYLY ELCVTAKSQT ECDIYPLRVG IRSVAVKGQQ FLINHKPFYF TGFGRHEDAD LRGKGFDNVL MVHDHALMDW
IGANSYRTSH YPYAEEMLDW ADEHGIVVID ETAAVGFNLS LGIGFEAGNK PKELYSEEAV NGETQQAHLQ AIKELIARDK
NHPSVVMWSI ANEPDTRPQG AREYFAPLAE ATRKLDPTRP ITCVNVMFCD AHTDTISDLF DVLCLNRYYG WYVQSGDLET
AEKVLEKELL AWQEKLHQPI IITEYGVDTL AGLHSMYTDM WSEEYQCAWL DMYHRVFDRV SAVVGEQVWN FADFATSQGI
LRVGGNKKGI FTRDRKPKSA AFLLQKRWTG MNFGEKPQQG GKQ

Sample 3

FHGSGIMVRP LNCIVAVSQN MGIGKNGDLP WPPLRNEFKY FQRMATTSSV EGKQNLVIMG RKTWFSIPEK NRPLKDRINI
VLSRELKEPP RGAHFLAKSL DDALRLIEQP ELASKVDMVW IVGGSSVYQE AMNQPGHLRL FVTRIMQEFE SDTFFPEIDL
GKYKLLPEYP GVLSEVQEDK GIKYKFEVYE KKDGSMGSYP YDVPDYAGYP YDVPDYAGSY PYDVPDYAGA GA...
MLRPVETPTR EIKKLDGLWA FSLDRENCGI DQRWWESALQ ESRAIAVPGS FNDQFADADI RNYAGNVWYQ REVFIPKGWA
GQRIVLRFDA VTHYGKVWVN NQEVMEHQGG YTPFEADVTP YVIAGKSVRI TVCVNNELNW QTIPPGMVIT DENGKKKQSY
FHDFFNYAGI HRSVMLYTTP NTWVDDITVV THVAQDCNHA SVDWQVVANG DVSVELRDAD QQVVATGQGT SGTLQVVNPH
LWQPGEGYLY ELCVTAKSQT ECDIYPLRVG IRSVAVKGQQ FLINHKPFYF TGFGRHEDAD LRGKGFDNVL MVHDHALMDW
IGANSYRTSH YPYAEEMLDW ADEHGIVVID ETAAVGFNLS LGIGFEAGNK PKELYSEEAV NGETQQAHLQ AIKELIARDK
NHPSVVMWSI ANEPDTRPQG AREYFAPLAE ATRKLDPTRP ITCVNVMFCD AHTDTISDLF DVLCLNRYYG WYVQSGDLET
AEKVLEKELL AWQEKLHQPI IITEYGVDTL AGLHSMYTDM WSEEYQCAWL DMYHRVFDRV SAVVGEQVWN FADFATSQGI
LRVGGNKKGI FTRDRKPKSA AFLLQKRWTG MNFGEKPQQG GKQ

SUPPLEMENTARY TABLES

Supplementary Table 1. Phenotypes of transgenic *Arabidopsis* plants in T1 generation.

Construct	No. of lines tested at 15°C possible ts-phenotype yes/no	No. of lines tested at 28°C possible ts-phenotype yes/no	scored as functional, i.e. ts (according to phenotypes at 16°C and 28°C)
K1:TTG1[1]	46/114	209/60	no
K2:TTG1[1]	**4/38**	**80/3**	**yes**
K3:TTG1[1]	2/10	10/3	no
K4:TTG1[1]	38/16	35/14	no
K1:CO[1]	30/190	169/16	no
K2:CO[1]	**2/10**	**11/0**	**yes**
K3:CO[1]	5/13	6/8	no
K4:CO[1]	3/8 *	n.a.	no (according to phenotype at semi-permissive temperature)
K1:TTG1-without linker[2]	13/12	19/6	no
K2:TTG1-without linker[2]	1/8	9/0	no
K3:TTG1- *without linker*[2]	**2/34**	**36/0**	**no**

This data is in correlation with phenotypic and protein data of **Supplementary Figure 3**. Exclusively K2:TTG1 and K2:CO, harboring both the identical K2 degron cassette (contains the short linker), were judged as functional because only here, both the conditional but also reversible phenotypes expected from a temperature-dependent system were observed. K2:TTG1 and K2:CO, both lacking the linker, are non-responding and do not show temperature-dependent phenotypes. K3:TTG1 without the linker was not scored as responsive because at cold temperature, only 2 out of 36 lines showed trichomes. The K3:TTG1 phenotypes were not reversible.

[1]T1 seeds were split into two pools and grown at either 16°C or 29°C, selected for presence of the transgene and phenotypically evaluated; * scored at semi-permissive temperature of 18-20°C.
[2]T1 seeds were first grown at 16°C, selected for presence of the transgene and phenotypically evaluated, then shifted to 29°C, and reevaluated.

Supplementary Table 2. Prediction of stability effects of K1 to K3 point mutations in murine DHFR.

**PoPMuSiC**[1]			
**Residue**	**Substitution**	**Properties**	**Predicted ΔΔG (kcal/mol)**
Thr39	n.a.	weakly stabilizing residue	-0.77
Thr39	T39A	destabilizing	> 0
Pro67	n.a.	n.a.	n.a.
Pro67	P66L	destabilizing	> 0
Glu173	n.a.	strongly stabilizing residue	-4.03
Glu173	E172D	destabilizing	> 0
**CUPSAT**[2]			
**Residue**	**Substitution**		
Thr39	n.a.	8 out of 19 possible substitutions (G, P, S, Q, K, Y, D, R) predicted to be destabilizing, but not T39A	
Thr39	T39A	stabilizing mutation, torsion unfavorable	1.39 (eighth highest value)*
Pro67	n.a.	all possible substitutions predicted to be destabilizing	
Pro67	P66L	destabilizing mutation, torsion unfavorable	-1.82 (twelfth lowest value)*
Glu173	n.a.	all possible substitutions predicted to be destabilizing	
Glu173	E172D	destabilizing mutation, torsion unfavorable	-2.07 (seventh lowest value)*
**PREDBUR and TSpred**[3]			
Thr39	n.a.	predicted to be unfavorable for introducing destabilizing mutations	n.a.
Thr39	T39A	not predicted to lead to instability	n.a.
Pro67	n.a.	predicted to be unfavorable for introducing destabilizing mutations	n.a.
Pro67	P66L	not predicted to lead to instability	n.a.
Glu173	n.a.	predicted to be unfavorable for introducing destabilizing mutations	n.a.
Glu173	E172D	not predicted to lead to instability	n.a.

* compared to remaining possible substitutions

[1] (Varadarajan et al., 1996; Chakshusmathi et al., 2004; Tan et al., 2014)

[2] (Parthiban et al., 2006)

[3] (Dehouck et al., 2011)

Supplementary Table 3. Plant and yeast strains used.

Strain	Genotype	Derivative of	reference
Arabidopsis			
Col-0	wildtype, accession Columbia-0	n.a.	NASC, The European Arabidopsis Stock Centre, NASC ID N1093 or N6673 or Lehle Seeds, Cat. No. WT-02, Arabidopsis genome sequencing project strain
ttg1	hit in At5g24520, T-DNA used: pGABI1, see www.gabi-kat.de for further details	Col-0	GABI_580A05, NASC stock ID: N455589,[3]
ttg1-13	deletion line from fast-neutron bombardment	Col-0	originally isolated from David Oppenheimer [University of Alabama, Tuscaloosa])[4]
S. cerevisiae			
JD47-13C	*MAT**a** his3-Δ200 leu2-3,112 lys2-801 trp1-Δ63 ura3-52*	-	[1]
JD53	*MATα his3-Δ200 leu2-3,112 lys2-801 trp1-Δ63 ura3-52*	JD47-13C	[5]
JD59	*MAT**a** ump1-Δ1::HIS3*	JD47-13C	[6]
JH5	*MAT**a** leu2-3,112 ura3-53* P_{GAL1}-*UMP1*	-	[6]

**Supplementary Table 4.** Oligonucleotide primers for cloning.			
**Construct**	**No.**	**name**	**Sequence (5' – 3')**
for degron cassettes only			
K1, K2, K3	ND70	ss12attB1UbcoreN	GGGGACAAGTTTGTACAAAAAAGCAGGCTTCCTCGAGCTG-CAGAATTACTATTTAC
K1	ND71	as1UbcoreCDHFRovlpN	GATGCCGGATCCGTGGAACCCACCTCTAAGTCTTAAGACAAG
K1	ND72	ss1DHFRcoreNUbovlpC	CTTAGAGGTGGGTTCCACGGATCCGGCATCAT
K1	ND73	as1DHFRovlpCHATcoreN	GTAGGATCCCATGGTACCGTCTTTCTTCTCGT
K1	ND74	ss1HATcoreNovlpDHFRC	GAAAGACGGTACCATGGGATCCTACCCATACGAT
K2, K3	ND78	as2UbcoreCDHFRovlpN	GATGCAGTTCAATGGTCGAACCATGATTCCAGATCCGTG-GAACCCACCTCTAAGTCTTAAGACAAG
K2, K3	ND79	ss2DHFRcoreNUbovlpC	CTTAGAGGTGGGTTCCACGGATCTGGAATCATGGTTCGACCATT-GAACTG
K2, K3, K4	ND80	as2DHFRovlpCHATcoreN	GTAGGATCCCATAGAACCGTCTTTCTTCTCGT
K2, K3, K4	ND81	ss2HATcoreNovlpDHFRC	GAAAGACGGTTCTATGGGATCCTACCCATACGAT
K2	N158	repK2ss	CTGGTTCTCCATTCCGGAGAAGAATCGACC
K2	N159	repK2as	GGTCGATTCTTCTCCGGAATGGAGAACC
K3	ND68	ss_P67L	CCATTCCTGAGAAGAATCGACTTTTAAAGGACCGAATTAATATAG
K3	ND69	as_P67L	CTATATTAATTCGGTCCTTTAAAAGTCGATTCTTCTCAGGAATGG
K4	ND82	ss4attB1DHFRcoreN	GGGGACAAGTTTGTACAAAAAA-GCAGGCTTCAACAATGGTTCGACCATTGAACTG
for reporter fusions			
TTG1			
K1, K4 (TTG1 constructs)	N172	asHATNotTTG1	CTGAATTATCCATACCAGCGGCCGCACCAGCGTAATCTGGAAC-GTCGTATG
K1, K4 (TTG1 constructs)	N173	ssHATNotTTG1	GTTCCAGATTACGCTGGTGCGGCCGCTGGTATGGA-TAATTCAGCTCCAGAT
K2, K3 (TTG1 constructs)	ND75	as1234HATcoreCovlpTTG	CTGAATTATCCATAGCACCAGCACCAGCGTAATCTGGAACGTCG-TATG
K2, K3 (TTG1 constructs)	ND76	ss1234TTGcoreNovlpHATC	GTTCCAGATTACGCTGGTGCTGGTGCTATGGA-TAATTCAGCTCCAGAT
K1, K2, K3, K4 (TTG1 constructs)	ND77	as1234TTGcoreC_attB2	GGGGACCACTTTGTACAAGAAA-GCTGGGTCTCAAACTCTAAGGAGCTGCAT
CO			
K1, K2, K3, K4 (CO con-structs)	N138	asHATNotCO	CTCTCTTGTTTCAACATACCAGCGGCCGCACCAGCGTAATCTG-GAACGTCGTATG
K1, K2, K3, K4 (CO con-structs)	N139	ssHATNotCO	GTTCCAGATTACGCTGGTGCGGCCGCTGGTATGTTGAAACAAGA-GAGTAACG
K1, K2, K3, K4 (CO con-structs)	N140	asCOattB2	GGGGACCACTTTGTACAAGAAAGCTGGGTCTCAT-ATCAGAATGAAGGAAC
GUS			
K2	TR08	asGUS-HAT	GGGGTTTCTACAGGACGTAACATAGCACCAGCACCAGCG-TAATCTGGAAC
	TR07	ssHAT-GUS	GTTCCAGATTACGCTGGTGCTGGTGCTATGTTACGTCCTG-TAGAAACCCC

	TR06	asattB2+GUS	GGGGACCACTTTGTACAAGAAAGCTGGGTCTTATTGTTTGCCTCCC
PAT			
K2	TR11	asPAT-HAT	GCCGGGCGTCGTTCTGGGCTCATAGCACCAGCACCAGCG-TAATCTGGAAC
	TR10	ssHAT-PAT	GTTCCAGATTACGCTGGTGCTGGTGCTATGAGCCCAGAACGAC-GCCCGGC
	TR09	asattB2+PAT	GGGGACCACTTTGTACAAGAAAGCTGGGTCTCAGATTTCGGTGAC-GGGCAGGACCGG
TEV			
K2	41	td-fwd	GGGGACAAGTTTGTACAAAAAAGC
	36	L4-HArev	CGCTCATGGGGTGATGGTGATGGTGATGTTTCATAGCGTAATCTG-GAACGTCGTATG
	46	LINKER2FORWARD	ATCACCATCACCCCATGAGCGGCCTGGTGCCGCGCGGCAGCGCC
	29	TEVREVERSE/TEVrev	TTACCCTTGCGAGTACACCAATTCA
without linker constructs			
	TR01	ssUbOverlapDHFR	CTTGTCTTAAGACTTAGAGGTGGGTTCGTTCGACCATTGAAC
	TR02	asDHFRoverlapUb	GCAGTTCAATGGTCGAACGAACCCACCTCTAAG
Drosophila			
K2:TEV	9	Ub-fwd	CACCATGCAGATTTTCGTCAAGACTTTGAC
	39	F-DHFR-UBrev	CCATGATTCCAGATCCGTGGAAACCACCTCTTAGCCTTAGCAC
	40	M-DHFR-UBrev	CCATGATTCCAGATCCGTGCATACCACCTCTTAGCCTTAGCAC
	37	F-DHFR	TTCCACGGATCTGGAATCATGG
	38	M-DHFR	ATGCACGGATCTGGAATCATGG
	36	L4-HArev	CGCTCATGGGGTGATGGTGATGGTGATGTTTCATAGCGTAATCTG-GAACGTCGTATG
	46	LINKER2FORWARD	ATCACCATCACCCCATGAGCGGCCTGGTGCCGCGCGGCAGCGCC
	29	TEVREVERSE/TEVrev	TTACCCTTGCGAGTACACCAATTCA
K2-ENTRY (pEN-L1-K2-L2)	21	K2-Pos2_frw	GCTGCCGCCATGGGAGGGGACAAGTTTGTACAA
	22	K2-Pos2_rw	GGGACCACTTTGTACAAGAAAGCTGGGTAGGCGCTGCCGCGCGG-CA

Supplementary Table 5. Oligonucleotide primers for sequencing.

Construct K	No.	name	Sequence (5' – 3')
1, 2, 3, 4	ND94	as_seqDHFR_N	CACGGCGACGATGCAGTTCAATGG
1, 2, 3, 4	ND95	ss_seqTTG_C	AGGCTAGTGTGAATGCTATAGC
1, 2, 3, 4	ND96	ss_seq_ovlpDHFR-TTG	CATCAAGTATAAGTTTGAAGTC
1, 2, 3, 4	N114	as_seqDHFR_N2	GACGGCGGTTTCCGATCTGGATAAC
1, 2, 3, 4	N122	ss_seqDHFR_N3	GACACGTTTTTCCCAGAAATTG
1, 2, 3, 4	N147	revCO	GAACCTCTGAATCACAGGCTGTGCATAG
1, 2, 3, 4	N148	fwdCO	GAACGCCCAAAGGGACAGTAG
prt1	N130	PRTpolyss	CAGAGGAAGAGCAAGAACGAGAAT
prt1	N131	PRTpolyas	CCACCTTCTGTTTATCTACAC
prt1	N143	ssPRT2	GATTATGTGGTTGCTTCTTGTGC
prt1	N144	asPRT2	GAAAGTTTTCCTCCAAAAGCTG

Supplementary Table 6. Antibodies used in this study.

antigen	risen in	name	cat. no.	supplier	used for
1° antibodies					
hemagglutinin epitope tag	mouse monoclonal	Anti-HA.11 Epitope Tag, Mouse IgG1, Clone: 16B12 (Covance; raw ascites fluid, not the purified version)	MMS-101R-1000	BioLegend or HISS	western blot 1:1000 dilution in TBST 5% milk
hemagglutinin epitope tag	mouse monoclonal	High Affinity, clone 3F10	11 867 423 001	Roche Diagnostics	western blot 1:1000 dilution in TBST 5% milk
PSTAIRE peptide epitope from Cyclin-dependent kinases of the Cdk1/2 type	rabbit polyclonal	Cdc2 p34 (PSTAIRE)	sc-53	Santa Cruz Biotechnol-ogy	western blot 1:1000 dilution in TBST 5% milk
Green fluorescent protein (GFP)	rabbit polyclonal	GFP (FL)	sc-8334	Santa Cruz Biotechnol-ogy	western blot 1:1000 dilution in TBST 5% milk
Green fluorescent protein (GFP)	mouse monoclonal	GFP (B-2)	sc-9996	Santa Cruz Biotechnol-ogy	western blot 1:1000 dilution in TBST 5% milk
human dehydrofolate reductase (DHFR)	mouse monoclonal	DHFR (A-4)	sc-74593	Santa Cruz Biotechnol-ogy	western blot 1:1000 dilution in TBST 5% milk
Ubiquitin	mouse monoclonal	Ub (P4D1)	sc-8017	Santa Cruz Biotechnol-ogy	western blot 1:1000 dilution in TBST 5% milk
β-Glucuronidase (GUS)	rabbit polyclonal	Anti β-Glucuronidase Rabbit IgG (H+L) Fraction	A-5790	Molecular Probes	western blot 1:500 dilution in TBST 5% milk
α Tubulin	rabbit polyclonal	α Tubulin (H-300)	sc-5546	Santa Cruz Biotechnol-ogy	western blot 1:1000 dilution in TBST 5% milk
Cdc11	rabbit polyclonal	Cdc11 (y-415)	sc-7170	Santa Cruz Biotechnol-ogy	western blot 1:1000 dilution in TBST 5% milk
2° antibodies					
mouse	goat IgG	anti-mouse IgG-HRP	1858415	Pierce	western blot 1:2500 dilution in TBST 5% milk (1:5000 for anti-HA)
rabbit	goat IgG	anti-rabbit IgG-HRP	1858413	Pierce	western blot 1:2500 dilution in TBST 5% milk

rat	goat IgG	Anti-Rat IgG (whole molecule)-Alkaline Phosphatase	A8438	Sigma-Aldrich	western blot 1:2500 dilution in TBST 5% milk

Supplementary Table 7. Oligonucleotide primers for RT-PCR.

Construct	name	Sequence (5’ – 3’)
**degron fusions**		
degron cassette	DHFR_frw	CCATTGAACTGCATCGTCGC
degron cassette	DHFR_rev	GCCTTTGTCCTCCTGGACCTC
**controls**		
ELONGA-TION-FACTOR1	EF1ss	ATGCCCCAGGACATCGTGATTTCAT
ELONGA-TION-FACTOR1	EF1as	TTGGCGGCACCCTTAGCTGGATCA
ACTIN2	Act2ss	GGCTCCTCTTAACCCAAAGGC
ACTIN2	Act2as	CACACCATCACCAGAATCCAGC
pAMPAT backbone, bla	bla_as	GACACGGAAATGTTGAATAC
**cDNA synthesis**		
oligo(dT) primers*	CDSIII-NotIA	ATTCTA-GAGGCCGAGGCGGCCGCCATGTTTTTTTTTTTTTTTTTTTTTTTTTTTTTTVA
oligo(dT) primers*	CDSIII-NotIC	ATTCTA-GAGGCCGAGGCGGCCGCCATGTTTTTTTTTTTTTTTTTTTTTTTTTTTTTTVC
oligo(dT) primers*	CDSIII-NotIG	ATTCTA-GAGGCCGAGGCGGCCGCCATGTTTTTTTTTTTTTTTTTTTTTTTTTTTTTTVG
oligo(dT) primers*	CDSIII-NotIT	ATTCTA-GAGGCCGAGGCGGCCGCCATGTTTTTTTTTTTTTTTTTTTTTTTTTTTTTTVT

* contain 30 desoxythymidines and XbaI and NotI sites

SUPPLEMENTARY NOTES

Supplementary Note 1

Comparison of current methods for conditional protein degradation directly acting on target protein levels

The lt-degron is a notable addition to the portfolio of existing tools for modulation of protein function. First, the obsolete rational trial-and-error generation and testing of supposed ts mutant variants of a POI can be avoided. Second, many reported tools focus on regulating the concentration of POIs through manipulating synthesis or the conformation rather than stability of present POI fusions or that they rely on transcriptional control and therefore is dependent on the intrinsic half-lives of inducers, mediators and targets. The lt-degron allows manipulation of degradation rates and therefore impinges directly on the level of the POI activity or function. Third, the lt-degron is a modular approach that gives control over a wide variety of target POIs in a number of host systems, Forth, and perhaps most importantly, the lt-degron works reliably and reversibly in multicellular organisms across the kingdoms due to a universal induction mode via temperature.

Then, the lt-degron method is primarily about conditional, reversible and direct control of protein levels. So far, the "classical" heat-induced N-degron system[1] involving the K1 cassette harboring $DHFR^{P67L}$ has only been used in yeasts and cell culture to conditionally switch POIs and requires restrictive temperatures as high as 37°C to 42°C.[5, 7, 8, 9] The biggest caveat of this technology was to date that these temperatures are beyond the physiological ranges of many multicellular organisms, e.g. plants.[10, 11, 12, 13, 14, 15]

Alternative methods for conditional protein shut-off which directly act on the level of target protein degradation are still largely limited to cells in culture or yeast as unicellular eukaryotes.[16] State-of-the-art inducible approaches for protein destabilization are compared in the following and rely mainly on 1) portable or dormant degrons that can be activated post-

translationally, 2) small molecules that act as molecular glue between POI and components of the degradation machinery, 3) degradation-mediating nanobodies targeted against the POI or a fusion containing the POI, or 4) reversible reconstitution of a degron or the POI

Dormant degrons

TIPI (TEV protease-mediated induction of protein instability) is a two-component protein degradation strategy for yeast and employs a chimeric POI fusion containing a dormant N-end rule degron at an unexposed central position and an inducible TEV protease. TEV cleaves the POI fusion at a defined sequence and thereby exposes an N-degron at the neo-N-terminal of the POI.[17, 18] The time of response depends mainly on the method used for TEV induction. Modified TIPI versions use the hormone β-estradiol to initiate TEV transcription[19] or additional C-terminal destabilizing moieties.[20]

Small molecule-mediators

The destabilizing domain (DD) system relies on a protein fusion technique involving protein stabilization dependent on the addition of the small molecule Shield1, a derivative of the immunosuppressant drug rapamycin.[21] The portable tag targeting the entire fusion to the proteasome is a constitutively unstable mutant variant of human FK506 binding protein 12 (FKBP12). The technology has been applied in a wide range of cell cultures.[16] Seed germination and plant growth seem not to be affected by Shield1 treatment suggesting that it is not toxic to plants[22] but further studies elaborating the potential use in multicellular organisms are required. A modification of the DD-FKBP system is LID (ligand-induced degradation)-FKBP that uses Shield1 for the opposite effect, i.e. causing POI instability after addition to the growth medium.[23]

In a PROTACS (proteolysis/protein targeting chimeric molecules) approach, bivalent chimeric molecules mediate protein–protein interaction and target POIs and their fusions to a

Skp1–Cullin–F-box (SCF) E3 Ub ligase complex for ubiquitination and degradation. The F-box protein β-TRCP has been used in pioneering studies in cell culture together with Protac-1, a chimeric molecule consisting of a phosphopeptide which serves as E3 interaction interface and a small molecule ligand that enables interaction with the target POI. The ligand is used as a handle for then joining the two moieties.[24, 25, 26] Various POIs, selected ligands and E3 ligases may prefer different features of the interaction-mediating small molecules and a custom chemical synthesis is mandatory and likely to require a specific design for a particular small molecule ligand.

The auxin-inducible degron (AID)[27] is a protein depletion tool that requires the transformation or transfection of two independent transgenes and the exogenous addition of a small molecule. It relies on the response signaling to the plant hormone auxin (indole-3-acetic acid or IAA) including the natural mechanism of destruction of the auxin-responsive transcriptional repressor protein INDOLE-3-ACETIC ACID INDUCIBLE 17 (IAA17)[28] and binding of auxin to TRANSPORT INHIBITOR RESPONSE1 (TIR1), a subunit of an SCF E3 Ub ligase complex. In the course of application, IAA17 needs to be fused to the POI and the TIR1-auxin-IAA17-POI complex is recruited to the proteasome via the SCF complex. The system seems to be limited by possible cytotoxicity,[29] and it cannot be applied in multicellular organisms due to the difficulty of application of the small molecule hormone nor in plant cell cultures due to the hormonal nature of the agent. It can only be applied in systems allowing homologous recombination.

Also the portable JAZ1 degron from the plant transcriptional repressor JASMONATE ZIM-DOMAIN 1 (JAZ1) is a two-component system requiring co-transformation of the degradation-mediating F-box protein CORONATINE INSENSITIVE 1 (COI1) and is triggered by the potent plant hormone jasmonoyl-L-isoleucine (JA-Ile) that functions as a molecular glue between JAZ1 and COI1. Target POIs are also recruited to the proteasome by a SCF E3 Ub ligase complex.[30]

Another small molecule-mediated approach is the tagging of a GOI by integration of an *E. coli* DHFR (eDHFR) degron within the genome via homologous recombination. This leads to sensitivity after deprivation of the structurally stabilizing antibiotic trimethoprim and effective depletion of eDHFR-tagged POIs.[31] It can only be applied in systems allowing homologous recombination and involves addition of a small molecule stabilizer.

Small molecule-mediated protein degradation is a strategy to create “chemical knock-outs”, however, the chemicals need to be introduced into the intracellular system which flags its preferential use in cultured cells. Application of these techniques can be difficult in multi-cellular organisms due to the need of application and penetration of the small molecules.

Nanobodies

The deGradFP (degrade Green Fluorescent Protein) protein depletion strategy directly targeting POI protein levels works in cell cultures and *Drosophila*. It depends on the presence of two stably transformed transgenes, i.e. a GFP fusion to a POI and an inducible anti-GFP nanobody.[32] This degradation mediator is comprised of the F-box domain of *Drosophila* Slmb and a single-domain camel antibody fragment and is under control of a chemically inducible promoters. Thus, the nanobody mediating target degradation is dependent on its own half-life and remains active until it gets degraded itself which happens in an unregulated manner. It will be challenging to introduce reversibility into this system.

Two recent additions to the nanobody methods include transcriptionally controlled conditional systems. The one contains a modified recognition element, namely SPOP, replacing the generic ant-GFP nanobody and is directed against specific nuclear proteins.[33] Is was applied in cell culture and zebra fish embryos. The GFE3 system is based on a fusion between the E3 ligase RING domain of XIAP and the recombinant antibody-like protein GFP–GPHN.FingR (gephyrin.Fibronectin intrabodies generated with mRNA display).[34] FingR is derived from the fibronectin 10FNIII domain and binds to gephyrin with high affinity. The

nanobody is induced by addition of an ecdysone analog and temporary expression of GFE3 was shown to inhibit synapse grow in zebra fish embryos.

Conformational inactivation

The light-dependent LOV2-mODC degron consists of the photosensitive LOV2 (LIGHT OXYGEN VOLTAGE 2) domain of *Arabidopsis* PHOTOTROPIN1 (PHOT1) which is activated upon irradiation with blue light,[35] undergoes a conformational change unmasking the previously cryptic degron of mODC (mouse ornithine decarboxylase).[36]

The protein disruption technique using temperature-sensitive inteins[37, 38] involves conditionally splicing of chimaeric protein fusions. It depends on the challenging reconstitution of a functional protein from a synthetic POI consisting of two (or more) inactive POI fragments separated by intein sequences. Folding, stability, and solubility issues need to be taken into account and, most importantly, for each target, functional disruption sites have to be identified that guarantee reconstitution of functional POI.[39] In an example of a thermostable intein-modified xylanase, recovery of enzyme activity occurred after activation by splicing at >59°C and was found to be significantly below the wild type levels.[40] This is a very good example for a biotechnological application of inteins in downstream processing after having obtained plant cell lysates or protein extracts.

Several of these methods, if modified, have certainly the potential to allow the generation and use of conditionally active proteins in multiple multicellular systems. More details on conditional genetic techniques can be found in a recent review of our lab.[16]

Supplementary Note 2

Selection of metabolically unstable mutant DHFR variants in S. cerevisiae and generation of a low-temperature-controlled N-degron

In order to achieve lower restrictive temperatures, first, metabolically unstable DHFR variants were isolated after a random PCR mutagenesis of the wild-type *DHFR* sequence fused to the *URA3* reporter gene encoding *Orotidine 5'-phosphate decarboxylase* (*Ura3*). A plasmid expressing *DHFR-Ura3* from P_{CUP1} (*pJH10*) served as a template for an error prone PCR mutagenesis that amplified a fragment starting within P_{CUP1} and terminating within the 5' portion of *URA3*. The resulting PCR products were then used together with the large fragment of EcoRI + BamHI digested *pJH10* (lacking the DHFR sequence) to transform *S. cerevisiae.* Incorporation of mutated versions of *DHFR* and recircularization of the plasmids occurred by *in vivo* recombination.

S. cerevisiae transformants expressing mutant *DHFR-Ura3* proteins that were degraded by the proteasome were selected using strain *JH5* (*ura3-53 leu2-3,112* P_{GAL1}*-UMP1*). In this strain, the *UMP1* gene, which encodes a proteasome maturation factor required for normal proteasome biogenesis,[6] is controlled by the galactose-inducible P_{GAL1} promoter. When P_{GAL1} is repressed in glucose-containing media, the strain behaves as a proteasome-deficient *ump1*Δ mutant with proteolysis defects. After mutagenesis, transformants were screened for functionality of the *URA3* reporter by plating onto two different media: one medium without uracil and one with uracil in combination with the toxin precursor 5-fluoroorotic acid (FOA).[41] Yeast cells with an active *URA3* gene can survive without external supplement of uracil but also convert FOA to fluorodeoxyuridine, which is toxic to cells. All yeast cells with sufficiently instable *DHFR* variants, will not be able to survive without uracil but recover on FOA. Thus, we used glucose media lacking uracil to select clones expressing *DHFR-Ura3* fusion proteins with *Ura3* activity. On galactose media with FOA, in contrast, variants were selected in which *Ura3* activity was sufficiently low due to degradation by the UPS. This se-

lection resulted in the isolation of the strain carrying plasmid *pJH10*^{mutC2} containing the *DHFR*^{T39A,E173D} variant carrying the two point mutations *T39A* and *E173D* which was used to generate construct *K2* (**Supplementary Figure 2c**).

Supplementary Note 3

Molecular modeling, dynamics simulations, and stability predictions of temperature-sensitive *DHFR* variants

Molecular modeling of temperature-sensitive *DHFR* variants was performed **t**o identify the underlying molecular cause for the enhanced temperature-sensitivity of the *K2-DHFR*^{T39A,E173D}. This *K2* variant was compared to the classical *K1-DHFR*^{P67L} and we estimated and analysed the impact of the three relevant substitutions by prediction methods, molecular dynamics (MD) and constructed models based on the crystal structure (**Supplementary Table 2**, **Supplementary Figure 5**). By calculating root mean standard deviation (RMSD), we found enhanced molecular flexibility in the protein structure of all three substitutions (**Supplementary Figure 5a-c**), indicating that the mutations lead to increased thermolability and cause a higher intramolecular flexibility between the neighboring amino acid residues and even within entire domains of the degron-DHFR (**Supplementary Figure 5d**).

To further elucidate the molecular origin of increased thermolability in the variants *K2* and *K3*, we modeled the three different point mutations used in the DHFR variants onto the wild type structure of DHFR (PDB ID: 1U70; **Supplementary Figure 5**). By investigating side chain conformations, we found that *T39A* does not essentially alter the structure of DHFR in the close vicinity of the point mutation (**Supplementary Figure 5e**). Nevertheless, the MD simulations show that Lys69 becomes more flexible and possibly also accessible for ubiquitination (**Supplementary Figure 5f,g**). Testing the effect of the *E173D* mutation resulted in a higher flexibility and accessibility of Lys174, which was accompanied with conformational changes of the side chains of Arg29 and Lys33 (**Supplementary Figure 5h,i**).

None of the three here discussed single point mutations was predicted to lead to significant instability of the DHFR by various techniques[42, 43, 44, 45, 46] (**Supplementary Table 2**).

As modeling template, a mouse DHFR X-ray structure (PDB ID: 1U70) crystallized as a ternary complex with methotrexate and the cofactor NADPH was used.[47] Prior to modeling,

all co-crystallized ligands were removed. Subsequently, the “Protonate 3D” tool of Molecular Operating Environment (MOE; version 2012.10; Chemical Computing Group) was applied to add all hydrogen atoms. Based on the prepared structure amino acid residue mutations under study were introduced using also MOE. For all these structures molecular dynamics simulations for 5 ns were performed with YASARA (http://www.yasara.org/index.html)[48] using the AMBER03 force field. Periodic boundary conditions including an appropriate water box were applied. The entire system was neutralized (pH 7.0) by adding sodium and chlorine ions.[49]

Stability prediction of DHFR point mutations was performed with PoPMuSiC, CUPSAT, and TSpred which are computer-aided tools for the prediction of changes in protein stability upon point mutations an the rationale design of mutant proteins affected in their stability. PoPMuSiC (http://dezyme.com/) evaluates the changes in stability of a given protein under single-site mutations on the basis of the structure of the protein.[45] The readout is the free energy change ΔΔG per sequence position where negative values for ΔΔG indicate lower stability. CUPSAT (Cologne University Protein Stability Analysis Tool; http://cupsat.tu-bs.de/) uses amino acid-atom potentials and torsion angle distribution to assess the amino acid environment of the mutation site. In case of unfavorable torsion angles, the atom potentials may have higher impact on stability which results in a stabilizing mutation.[44] TSpred[46] based on PREDBUR predict the potential of point mutations based on their hydrophobicity and hydrophobic moment.[42, 43]

SUPPLEMENTARY REFERENCES

1. Dohmen R, Wu P, Varshavsky A. Heat-inducible degron: a method for constructing temperature-sensitive mutants. *Science* **263**, 1273-1276 (1994).

2. Gowda N, Kandasamy G, Froehlich M, Dohmen R, Andreasson C. Hsp70 nucleotide exchange factor Fes1 is essential for ubiquitin-dependent degradation of misfolded cytosolic proteins. *Proc Natl Acad Sci U S A* **110**, 5975-5980 (2013).

3. Kleinboelting N, Huep G, Kloetgen A, Viehoever P, Weisshaar B. GABI-Kat SimpleSearch: new features of the Arabidopsis thaliana T-DNA mutant database. *Nucleic Acids Res* **40**, D1211-1215 (2012).

4. Walker A, *et al.* The TRANSPARENT TESTA GLABRA1 locus, which regulates trichome differentiation and anthocyanin biosynthesis in Arabidopsis, encodes a WD40 repeat protein. *Plant Cell* **11**, 1337-1350 (1999).

5. Dohmen R, Varshavsky A. Heat-inducible degron and the making of conditional mutants. *Methods Enzymol* **399**, 799-822 (2005).

6. Ramos P, Hockendorff J, Johnson E, Varshavsky A, Dohmen R. Ump1p is required for proper maturation of the 20S proteasome and becomes its substrate upon completion of the assembly. *Cell* **92**, 489-499 (1998).

7. Su X, Bernal J, Venkitaraman A. Cell-cycle coordination between DNA replication and recombination revealed by a vertebrate N-end rule degron-Rad51. *Nat Struct Mol Biol* **15**, 1049-1058 (2008).

8. Kearsey S, Gregan J. Using the DHFR heat-inducible degron for protein inactivation in Schizosaccharomyces pombe. *Methods Mol Biol* **521**, 483-492 (2009).

9. Bernal J, Venkitaraman A. A vertebrate N-end rule degron reveals that Orc6 is required in mitosis for daughter cell abscission. *J Cell Biol* **192**, 969-978 (2011).

10. Gray W, Ostin A, Sandberg G, Romano C, Estelle M. High temperature promotes auxin-mediated hypocotyl elongation in Arabidopsis. *Proc Natl Acad Sci U S A* **95**, 7197-7202 (1998).

11. Larkindale J, Hall J, Knight M, Vierling E. Heat stress phenotypes of Arabidopsis mutants implicate multiple signaling pathways in the acquisition of thermotolerance. *Plant Physiol* **138**, 882-897 (2005).

12. Koini M, *et al.* High temperature-mediated adaptations in plant architecture require the bHLH transcription factor PIF4. *Curr Biol* **19**, 408-413 (2009).

13. Kumar S, Wigge P. H2A.Z-containing nucleosomes mediate the thermosensory response in Arabidopsis. *Cell* **140**, 136-147 (2010).

14. Franklin K, *et al.* Phytochrome-interacting factor 4 (PIF4) regulates auxin biosynthesis at high temperature. *Proc Natl Acad Sci U S A* **108**, 20231-20235 (2011).

15. Quint M, Delker C, Franklin KA, Wigge PA, Halliday KJ, van Zanten M. Molecular and genetic control of plant thermomorphogenesis. *Nature Plants* **2**, 15190 (2016).

16. Faden F, Mielke S, Lange D, Dissmeyer N. Generic tools for conditionally altering protein abundance and phenotypes on demand. *Biol Chem* **395**, 737-762 (2014).

17. Taxis C, Stier G, Spadaccini R, Knop M. Efficient protein depletion by genetically controlled deprotection of a dormant N-degron. *Mol Syst Biol* **5**, 267 (2009).

18. Taxis C, Knop M. TIPI: TEV protease-mediated induction of protein instability. *Methods Mol Biol* **832**, 611-626 (2012).

19. McIsaac R, *et al.* Fast-acting and nearly gratuitous induction of gene expression and protein depletion in Saccharomyces cerevisiae. *Mol Biol Cell* **22**, 4447-4459 (2011).

20. Jungbluth M, Renicke C, Taxis C. Targeted protein depletion in Saccharomyces cerevisiae by activation of a bidirectional degron. *BMC Syst Biol* **4**, 176 (2010).

21. Banaszynski L, Chen L, Maynard-Smith L, Ooi A, Wandless T. A rapid, reversible, and tunable method to regulate protein function in living cells using synthetic small molecules. *Cell* **126**, 995-1004 (2006).

22. Su L, Li A, Li H, Chu C, Qiu JL. Direct modulation of protein level in Arabidopsis. *Mol Plant* **6**, 1711-1714 (2013).

23. Bonger K, Chen L, Liu C, Wandless T. Small-molecule displacement of a cryptic degron causes conditional protein degradation. *Nat Chem Biol* **7**, 531-537 (2011).

24. Sakamoto K, Kim K, Kumagai A, Mercurio F, Crews C, Deshaies R. Protacs: chimeric molecules that target proteins to the Skp1-Cullin-F box complex for ubiquitination and degradation. *Proc Natl Acad Sci U S A* **98**, 8554-8559 (2001).

25. Schneekloth J, *et al.* Chemical genetic control of protein levels: selective in vivo targeted degradation. *J Am Chem Soc* **126**, 3748-3754 (2004).

26. Carmony K, Kim K. PROTAC-induced proteolytic targeting. *Methods Mol Biol* **832**, 627-638 (2012).

27. Nishimura K, Fukagawa T, Takisawa H, Kakimoto T, Kanemaki M. An auxin-based degron system for the rapid depletion of proteins in nonplant cells. *Nat Methods* **6**, 917-922 (2009).

28. Gray WM, Kepinski S, Rouse D, Leyser O, Estelle M. Auxin regulates SCF(TIR1)-dependent degradation of AUX/IAA proteins. *Nature* **414**, 271-276 (2001).

29. Folkes L, Dennis M, Stratford M, Candeias L, Wardman P. Peroxidase-catalyzed effects of indole-3-acetic acid and analogues on lipid membranes, DNA, and mammalian cells in vitro. *Biochem Pharmacol* **57**, 375-382 (1999).

30. Sheard L, *et al.* Jasmonate perception by inositol-phosphate-potentiated COI1-JAZ co-receptor. *Nature* **468**, 400-405 (2010).

31. Sheridan RM, Bentley DL. Selectable one-step PCR-mediated integration of a degron for rapid depletion of endogenous human proteins. *Biotechniques* **60**, 69-74 (2016).

32. Caussinus E, Kanca O, Affolter M. Fluorescent fusion protein knockout mediated by anti-GFP nanobody. *Nat Struct Mol Biol* **19**, 117-121 (2012).

33. Shin YJ*, et al.* Nanobody-targeted E3-ubiquitin ligase complex degrades nuclear proteins. *Sci Rep* **5**, 14269 (2015).

34. Gross GG*, et al.* An E3-ligase-based method for ablating inhibitory synapses. *Nat Meth* **advance online publication**, (2016).

35. Renicke C, Schuster D, Usherenko S, Essen L, Taxis C. A LOV2 domain-based optogenetic tool to control protein degradation and cellular function. *Chem Biol* **20**, 619-626 (2013).

36. Mills E, Truong K. Photoswitchable protein degradation: a generalizable control module for cellular function? *Chem Biol* **20**, 458-460 (2013).

37. Zeidler MP*, et al.* Temperature-sensitive control of protein activity by conditionally splicing inteins. *Nat Biotechnol* **22**, 871-876 (2004).

38. Tan G, Chen M, Foote C, Tan C. Temperature-sensitive mutations made easy: generating conditional mutations by using temperature-sensitive inteins that function within different temperature ranges. *Genetics* **183**, 13-22 (2009).

39. Sonntag T, Mootz H. An intein-cassette integration approach used for the generation of a split TEV protease activated by conditional protein splicing. *Mol Biosyst* **7**, 2031-2039 (2011).

40. Shen B*, et al.* Engineering a thermoregulated intein-modified xylanase into maize for consolidated lignocellulosic biomass processing. *Nat Biotechnol* **30**, 1131-1136 (2012).

41. Ghislain M, Dohmen R, Levy F, Varshavsky A. Cdc48p interacts with Ufd3p, a WD repeat protein required for ubiquitin-mediated proteolysis in Saccharomyces cerevisiae. *EMBO J* **15**, 4884-4899 (1996).

42. Varadarajan R, Nagarajaram H, Ramakrishnan C. A procedure for the prediction of temperature-sensitive mutants of a globular protein based solely on the amino acid sequence. *Proc Natl Acad Sci U S A* **93**, 13908-13913 (1996).

43. Chakshusmathi G*, et al.* Design of temperature-sensitive mutants solely from amino acid sequence. *Proc Natl Acad Sci U S A* **101**, 7925-7930 (2004).

44. Parthiban V, Gromiha M, Schomburg D. CUPSAT: prediction of protein stability upon point mutations. *Nucleic Acids Res* **34**, W239-242 (2006).

45. Dehouck Y, Kwasigroch J, Gilis D, Rooman M. PoPMuSiC 2.1: a web server for the estimation of protein stability changes upon mutation and sequence optimality. *BMC Bioinformatics* **12**, 151 (2011).

46. Tan K, Khare S, Varadarajan R, Madhusudhan M. TSpred: a web server for the rational design of temperature-sensitive mutants. *Nucleic Acids Res* **42**, W277-284 (2014).

47. Cody V, Luft J, Pangborn W. Understanding the role of Leu22 variants in methotrexate resistance: comparison of wild-type and Leu22Arg variant mouse and human dihydrofolate reductase ternary crystal complexes with methotrexate and NADPH. *Acta Crystallogr D Biol Crystallogr* **61**, 147-155 (2005).

48. Krieger E, Koraimann G, Vriend G. Increasing the precision of comparative models with YASARA NOVA--a self-parameterizing force field. *Proteins* **47**, 393-402 (2002).

49. Krieger E, Nielsen J, Spronk C, Vriend G. Fast empirical pKa prediction by Ewald summation. *J Mol Graph Model* **25**, 481-486 (2006).

Supplementary Figure 8.. Original uncropped images of gels, western blots and membranes

Figure 1

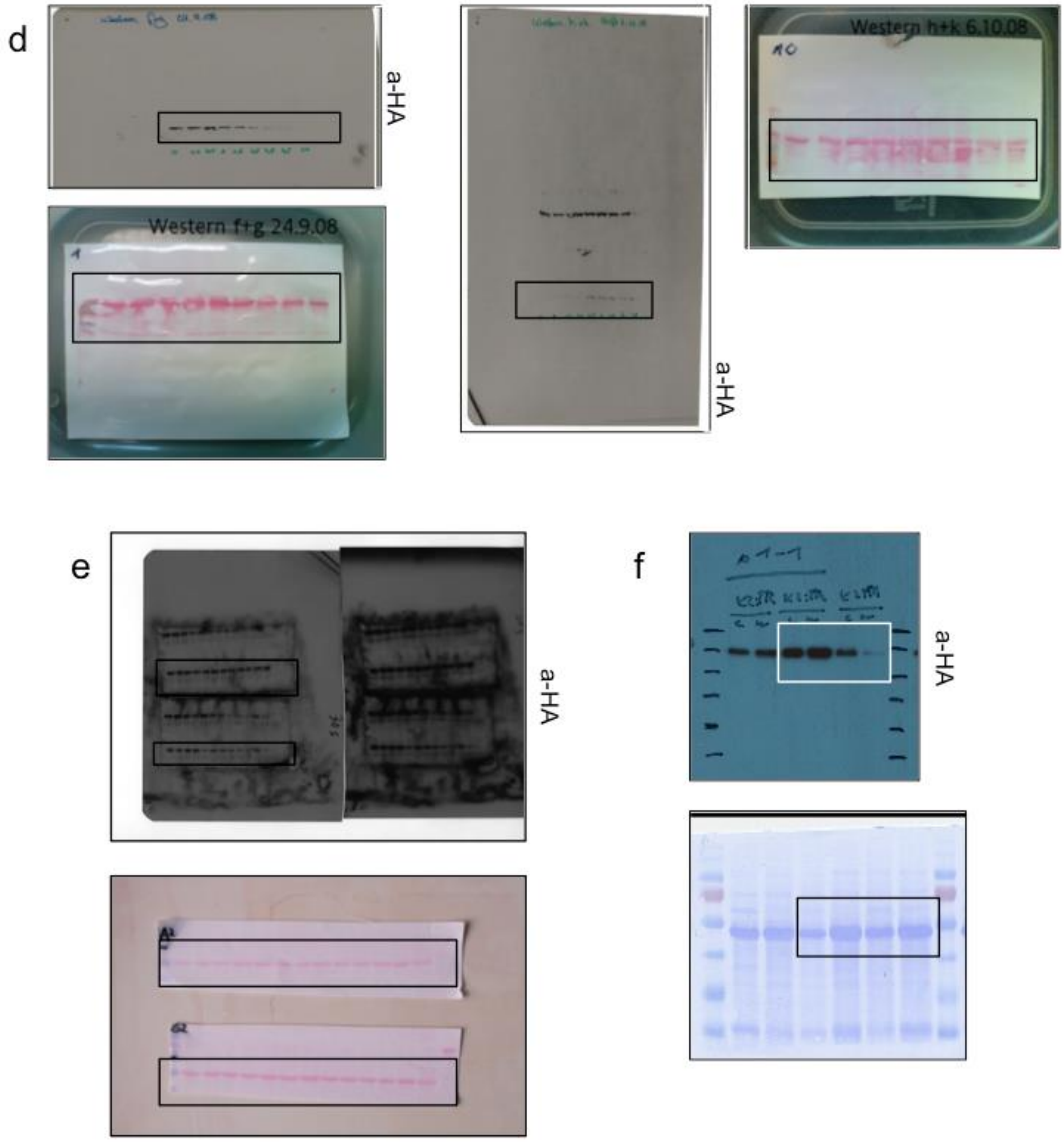

Figure 3

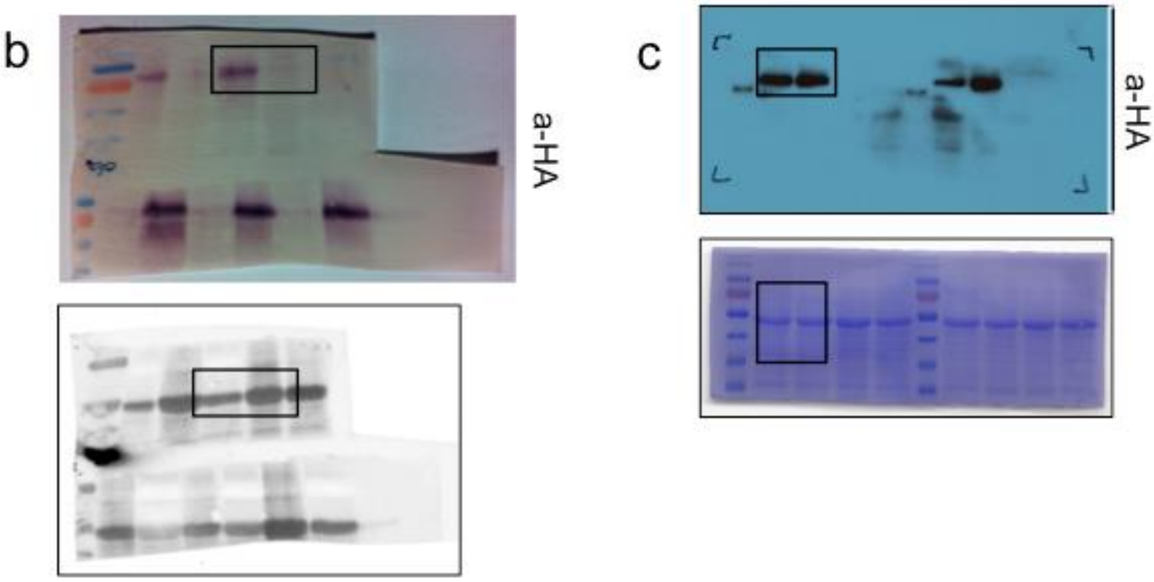

Figure 4

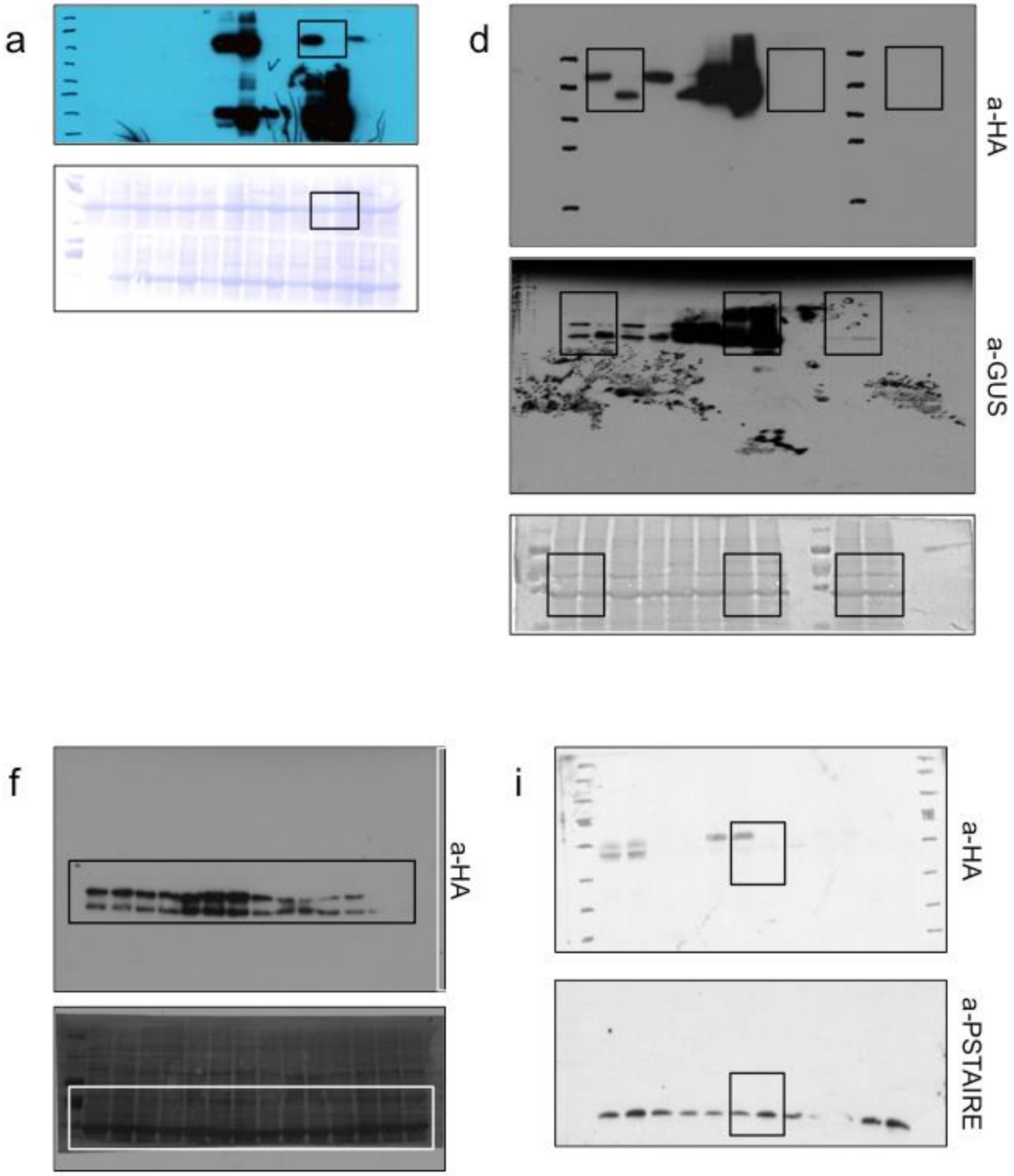

Figure 5

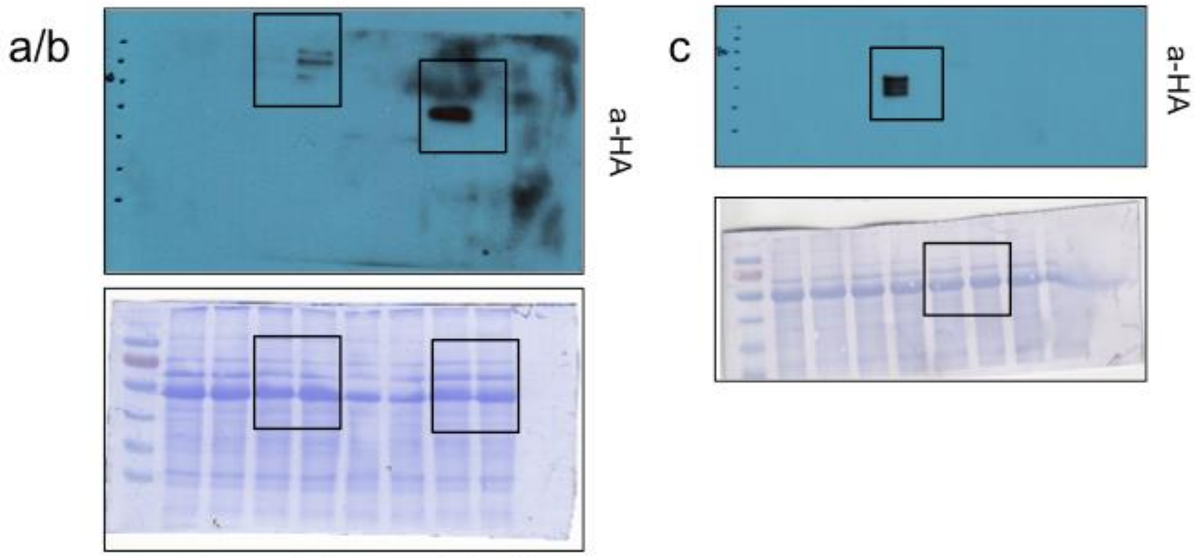

K2:GUS loaded inversely (w/C)

Figure 7

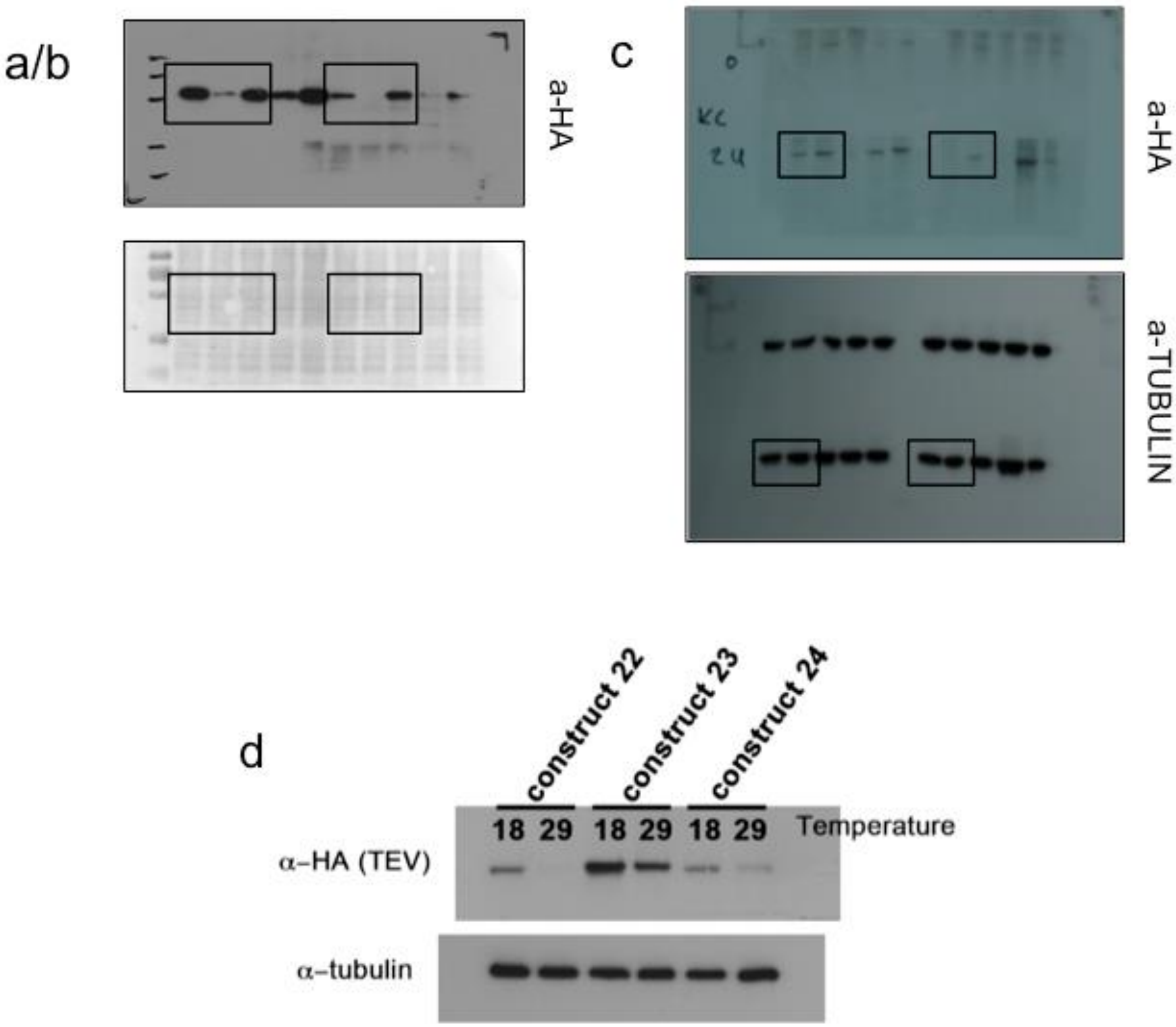
a/b
a-HA
c
a-HA
a-TUBULIN
d
construct 22
construct 23
construct 24
18 29 18 29 18 29
Temperature
α–HA (TEV)
α–tubulin

Supplementary Figure 3

a

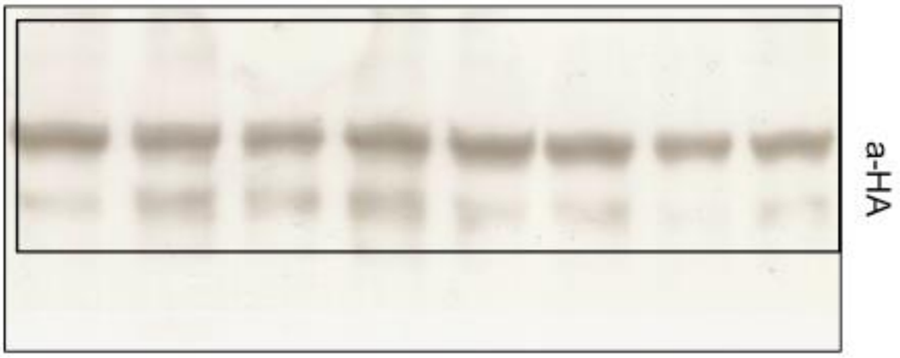

c

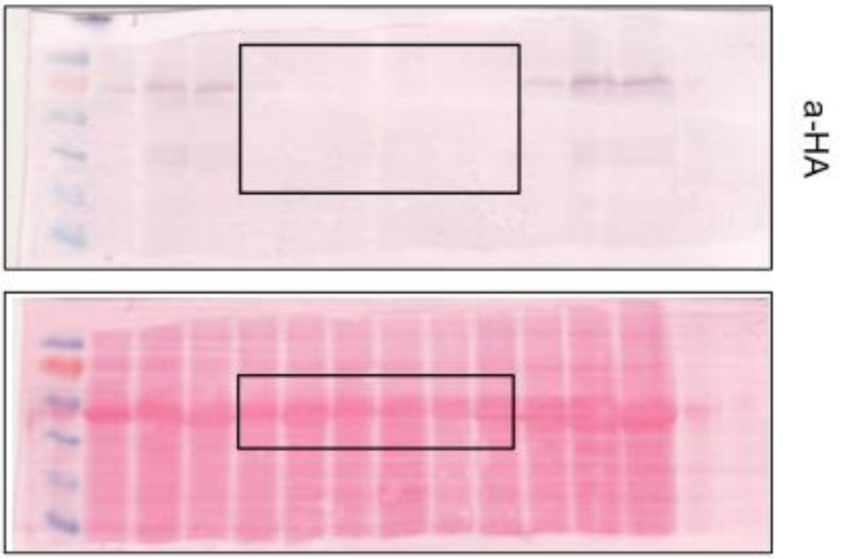

d

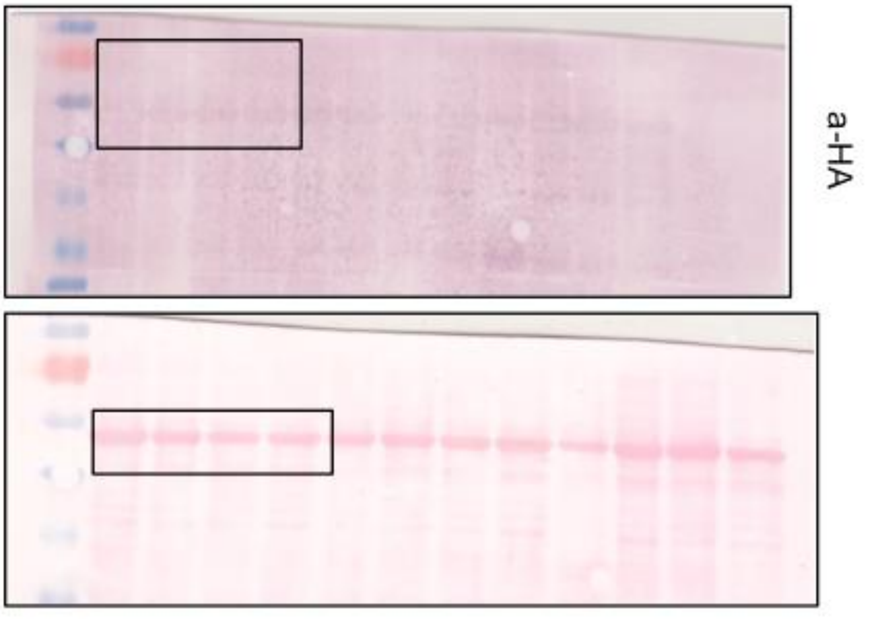

Supplementary Figure 4

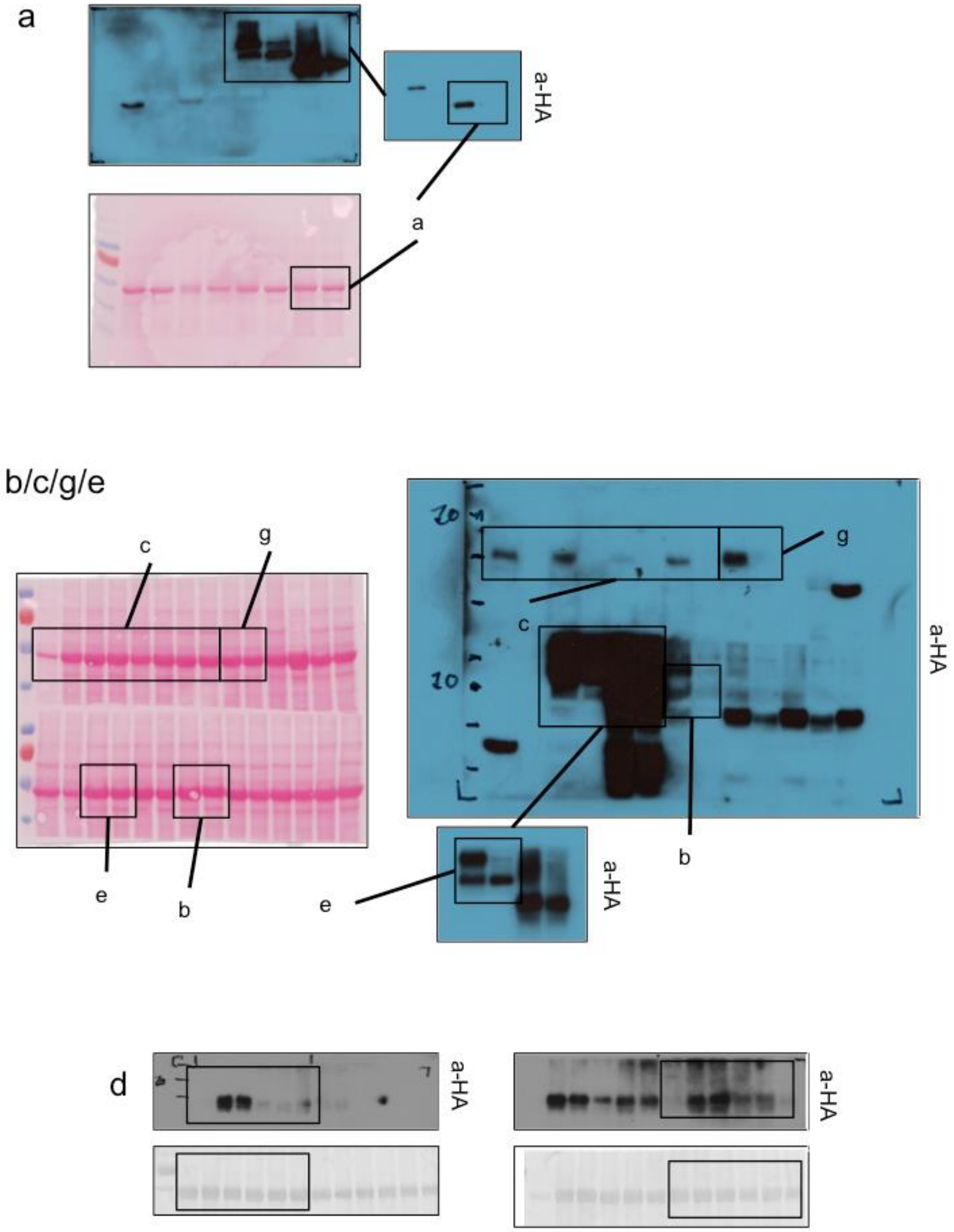

Supplementary Figure 4 (continued)

f

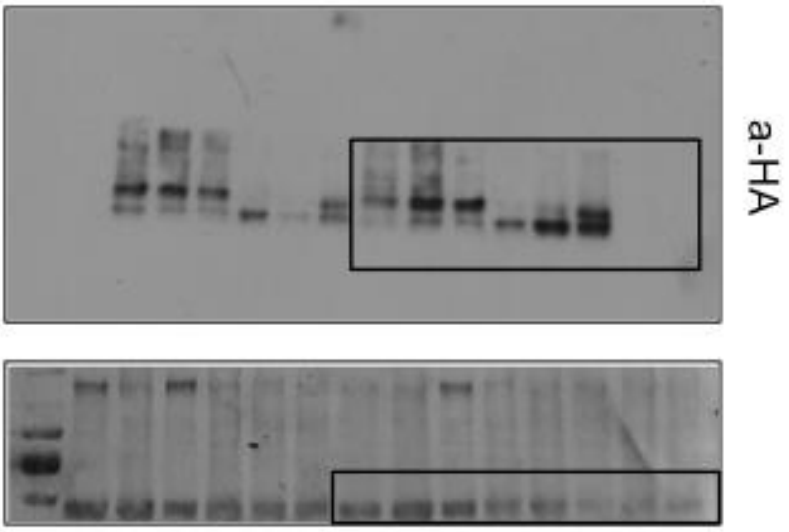

Supplementary Figure 7

a

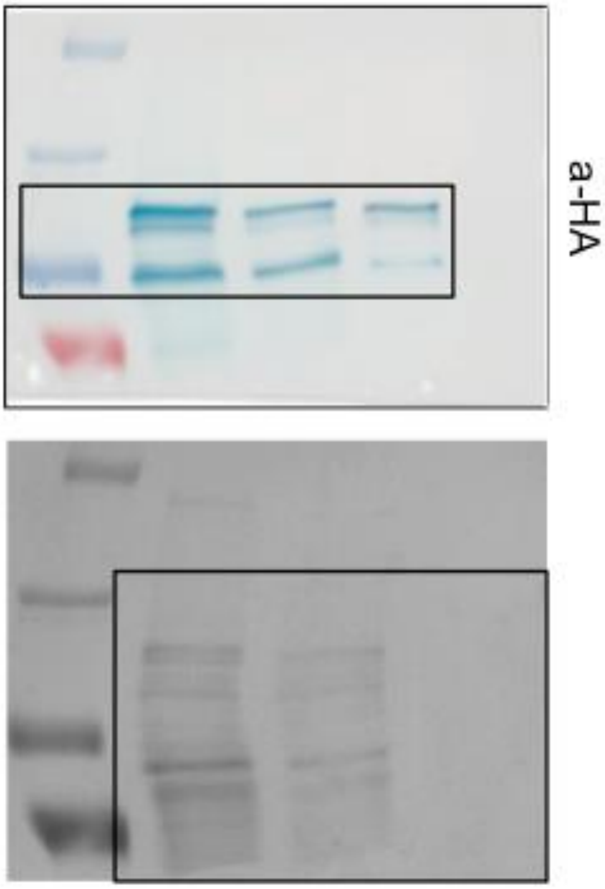

Supplementary Figure 3 and 4

RT-PCR *DHFR*

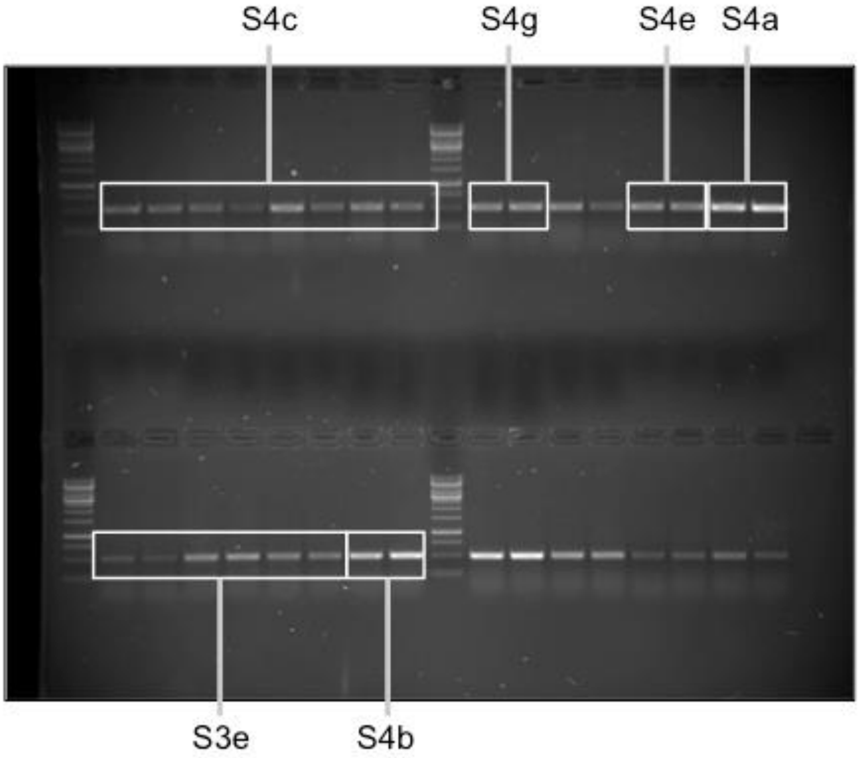

RT-PCR *EF1a*

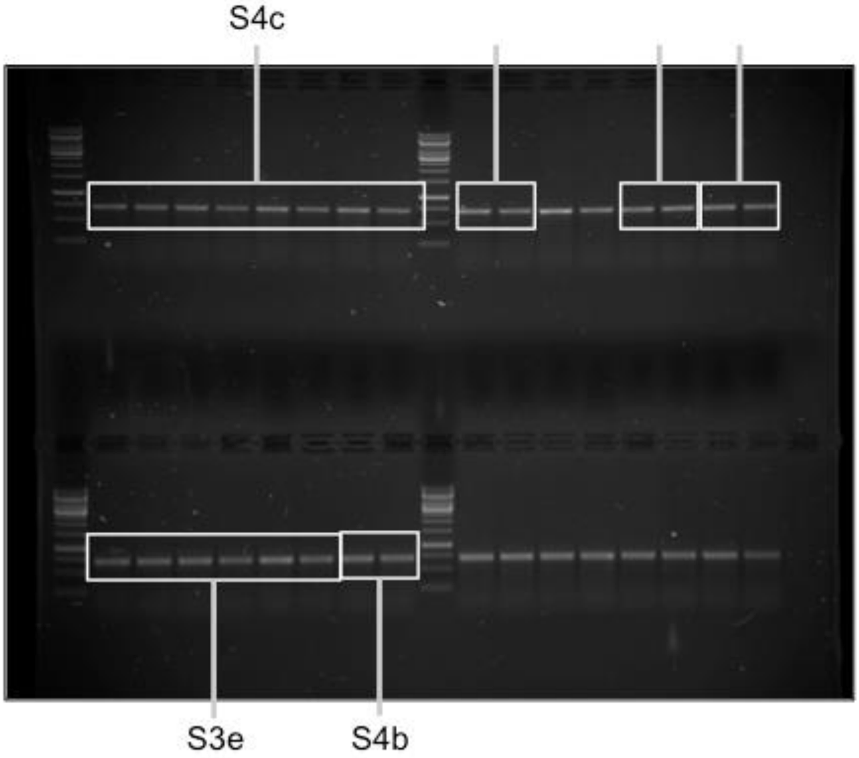

Supplementary Figure 3 and 4 (continued)

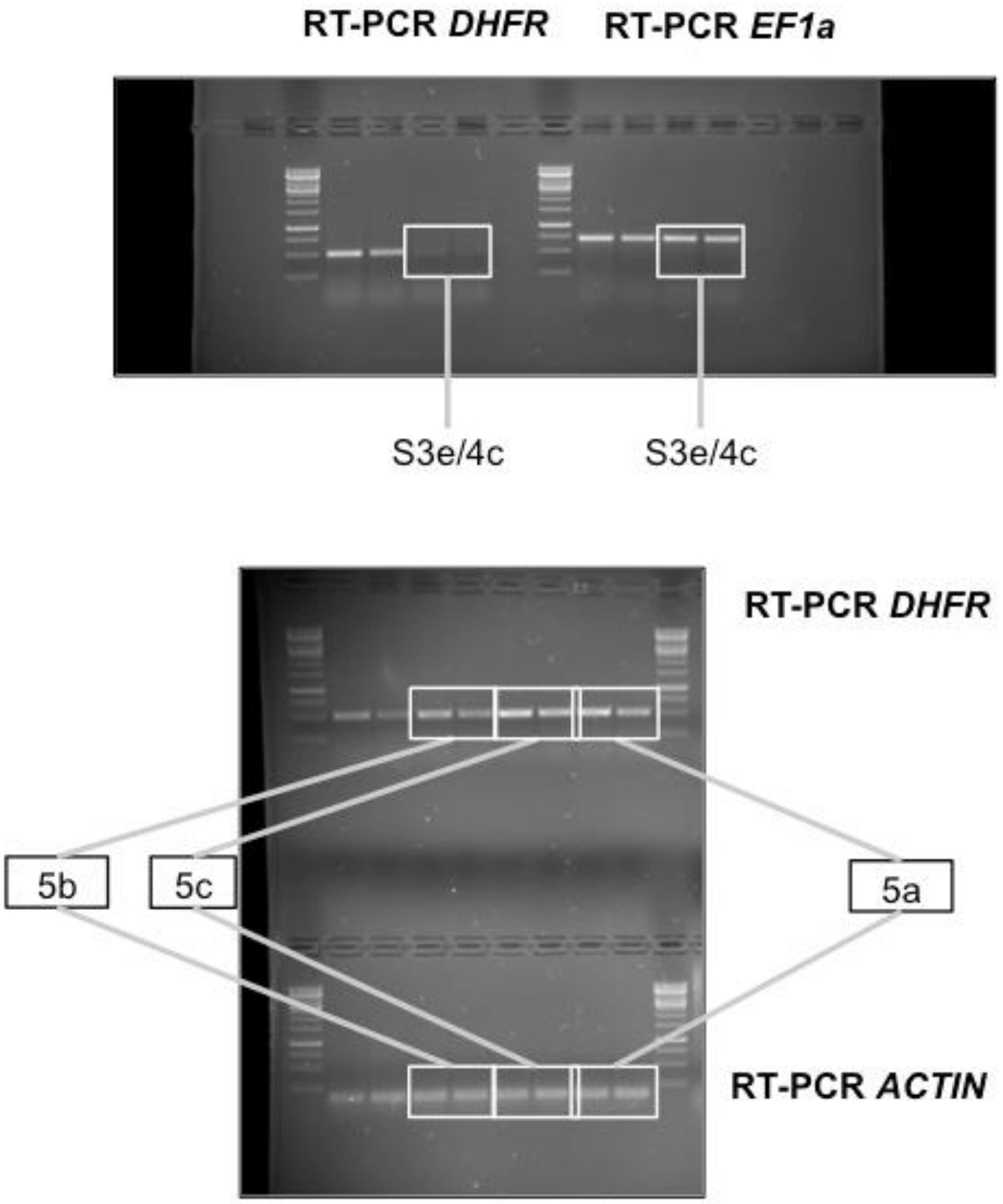

Supplement: Supplementary Information — Supplementary Figures 1 - 8, Supplementary Tables 1 - 7, Supplementary Notes 1-3 and Supplementary References [file ncomms12202-s1.pdf]
